# Supplementary material for: NAP-seq reveals multiple classes of structured noncoding RNAs with regulatory functions
Source: Nat Commun. 2024 Mar 18;15:2425. doi: 10.1038/s41467-024-46596-y (PMC10948791; doi:10.1038/s41467-024-46596-y)
Supplement: Supplementary file 1 — Supplementary information [file 41467_2024_46596_MOESM1_ESM.pdf]

Supplementary information for

# NAP-seq reveals multiple classes of structured noncoding RNAs with regulatory functions

**Authors:** Shurong Liu[1]†, Junhong Huang[1,2]†, Jie Zhou[1], Siyan Chen[1,2], Wujian Zheng[1], Chang Liu[1], Qiao Lin[1], Ping Zhang[1], Di Wu[1,2], Simeng He[2], Jiayi Ye[1], Shun Liu[3], Keren Zhou[4], Bin Li[1]*, Lianghu Qu[1]*, Jianhua Yang[1,2]*

Correspondence to: Jianhua Yang (Email: yangjh7@mail.sysu.edu.cn), Lianghu Qu (Email: lssqlh@mail.sysu.edu.cn), Bin Li, Email: libin73@mail.sysu.edu.cn

**This PDF file includes:**

Supplementary Figs. 1-20;

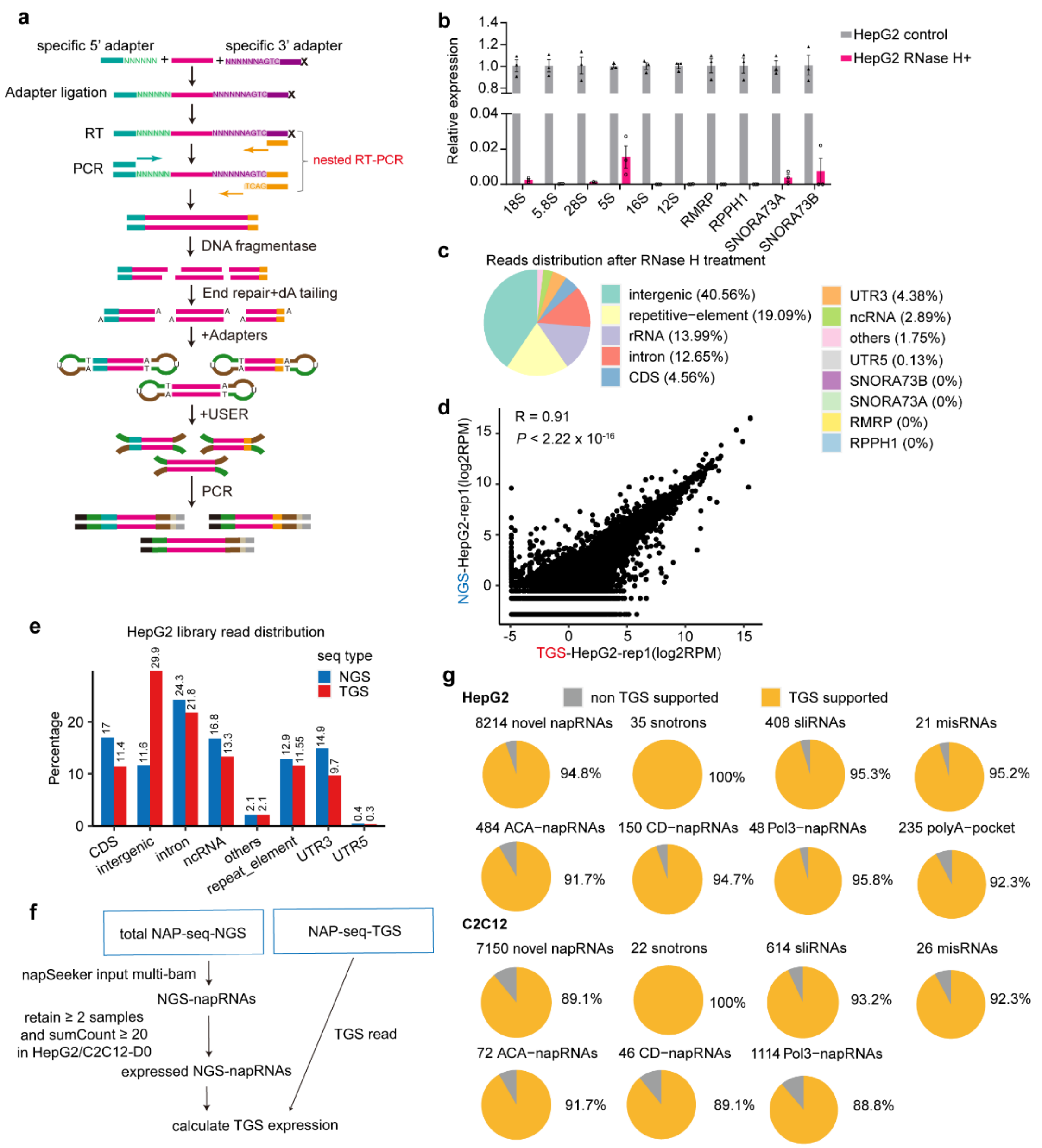


**Supplementary Figure 1. NAP-seq-NGS and NAP-seq-TGS exhibit a strong correlation. a** The design of dual RNA adapters for capturing both ends of napRNAs and the schematic of NAP-seq-NGS. **b, c** Removal efficiency of RNAs from total RNA using DNA probes and RNase H treatment. The expression level of rRNA and some snoRNAs were measured by RT-qPCR with RNase H treatment (**b**). Source data are provided as a Source Data file. Genomic distribution of reads in HepG2 rep1 library after RNase H treatment (**c**). **d** Scatterplot show the correlation between reads obtained by the NAP-seq-NGS and NAP-seq-TGS (rep1, replicate1). Read numbers, RPM, reads per million. *P* value was calculated by two-sided Pearson's correlation test. **e** The

distribution and the percentage of NAP-seq-NGS and NAP-seq-TGS reads in annotated gene types. **f** Workflow of napRNA identification in NAP-seq-NGS and NAP-seq-TGS. **g** Pie plot show that the napRNAs identified by NAP-seq-NGS were supported by NAP-seq-TGS in humans and mice.

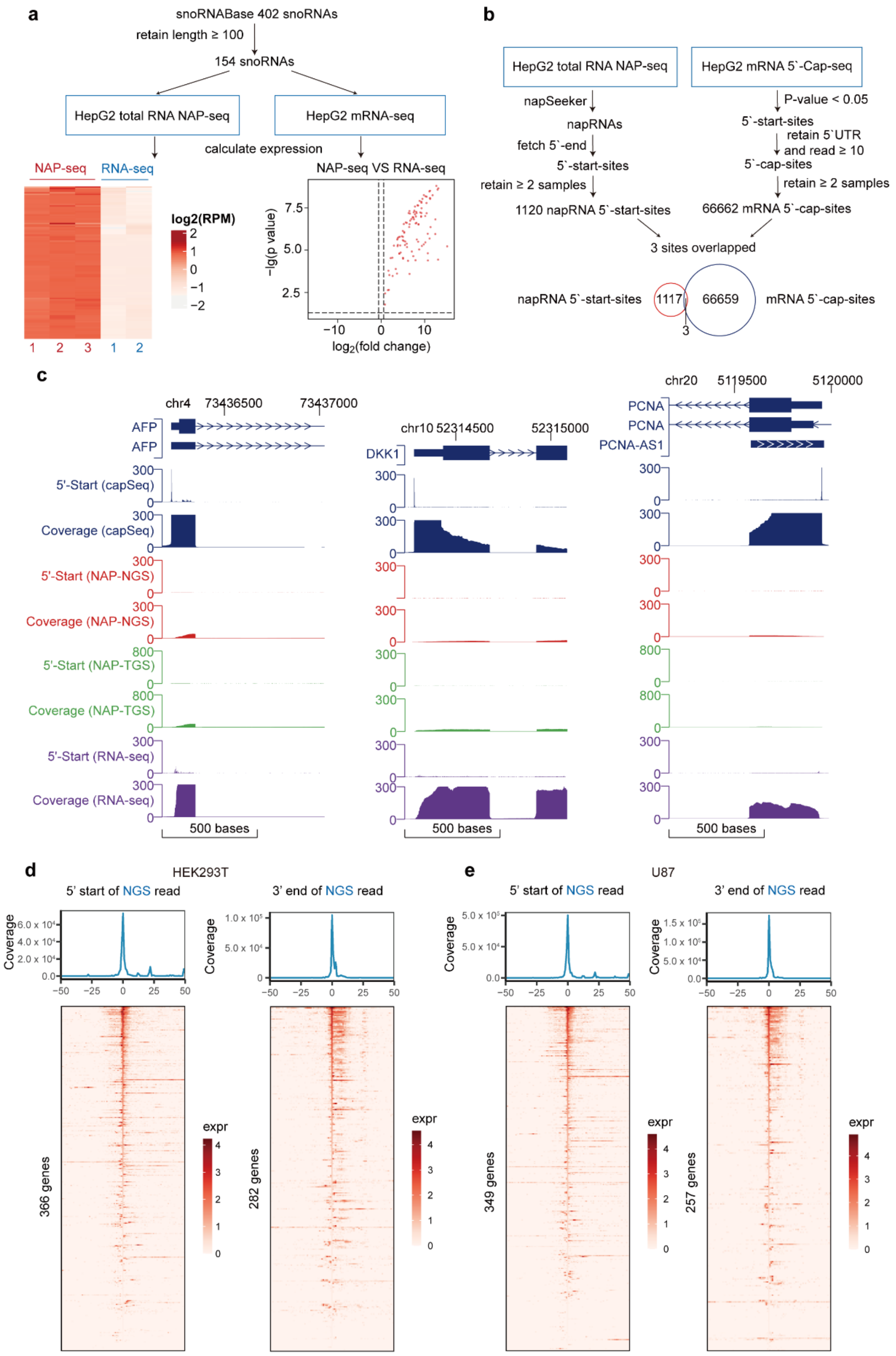


**Supplementary Figure 2. NAP-seq reliably detects napRNAs at single-nucleotide resolution.** **a** snoRNAs with >100nt length can be detected by NAP-seq and mRNA-seq. **b** Intersection of 5'-start-sites of napRNAs detected by NAP-seq and 5'-cap-sites detected by CAP-seq. **c** Genome Browser view of 5'-start signals from NAP-seq and

CAP-seq (RPM, reads per million) in an extended region of three overlapped sites. **d and e** Number of coincidences between the 5'-start (or 3'-end) sites in NAP-seq-NGS reads and the 5'-start (or 3'-end) sites in known ncRNAs in HEK293T (**d**) and U87 (**e**) cells. The x-axis shows the distance from the 5'-start (or 3'-end) site in a NAP-seq-NGS read to the annotated 5'-start (or 3'-end) sites in known ncRNAs, and the y-axis shows the number of reads within a certain distance. The bottom panel shows a heatmap, in which each row represents a gene that shares the same 5'-start (or 3'-end) site with the NAP-seq-NGS read, and each column represents the expression values of genes at a specific distance. expr, expression value.

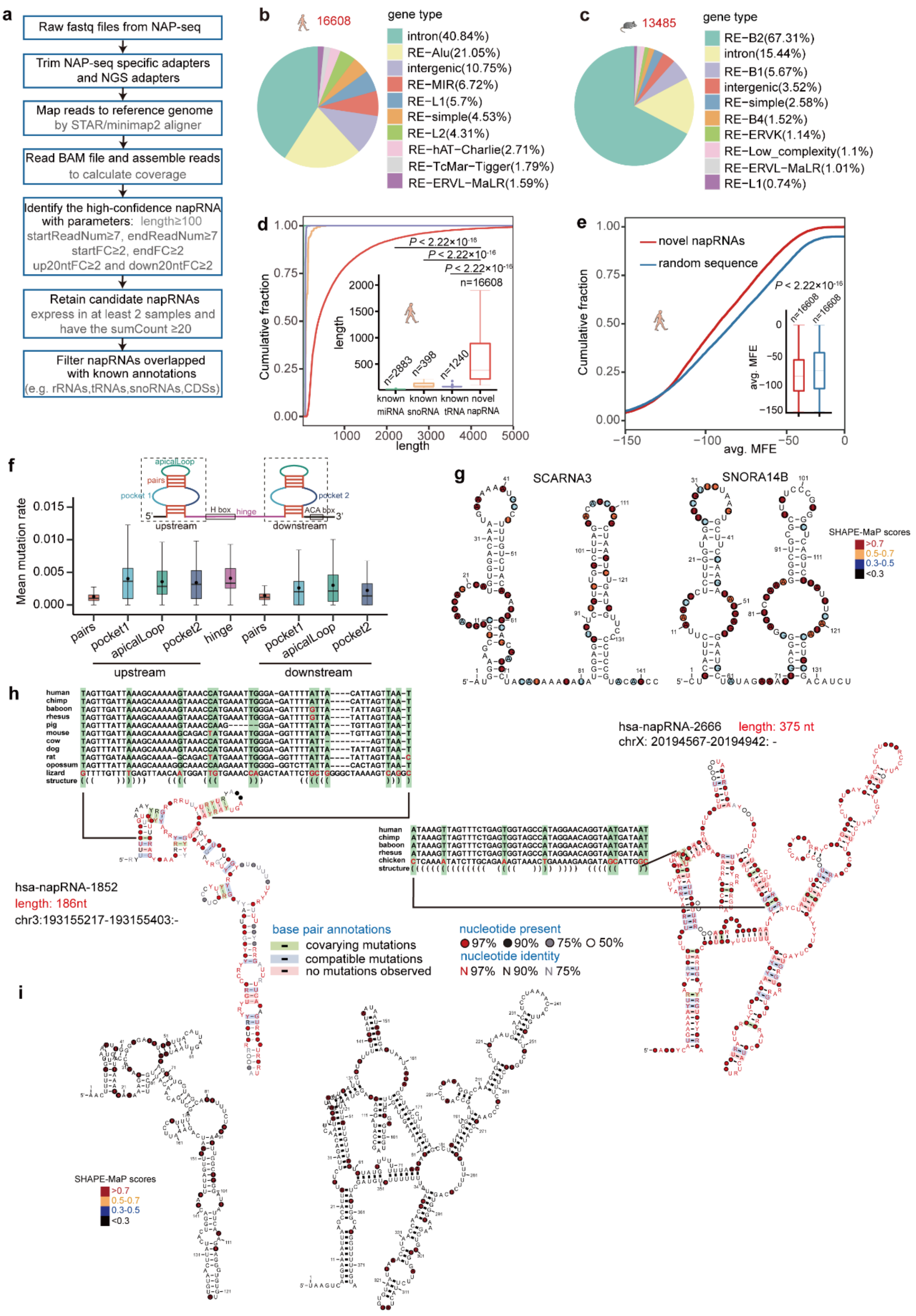


**Supplementary Figure 3. Characters of structured ncRNAs detected by NAP-seq.** **a** Overview of the computational pipeline applied for NAP-seq analysis. **b and c** Distribution of napRNAs identified by NAP-seq-NGS within annotated gene types in humans (**b**) and mice (**c**). RE, repetitive elements. **d** Cumulative curves and box plots

showing the lengths of previously undiscovered napRNAs identified by NAP-seq and known ncRNAs (including miRNAs, snoRNAs, and tRNAs) in humans. **e** Cumulative curves and box plots showing the average minimum free energy (avg. MFE) values of napRNAs identified by NAP-seq and of random sequences in humans. *P* values in **d** and **e** were calculated by two-sided Mann-Whitney-Wilcoxon test. Each boxplot shows the minima, maxima, centre, bounds of box, whiskers, first and third percentile. **f** NAP-SHAPE-MaP reactivity of the napRNA. X axis represent various secondary structure region of napRNA. Y axis represent the mean reactivity of SHAPE-MaP. **g** The secondary structure model and SHAPE-MaP reactivity score for each base of SCARNA3 (left) and SNORA14B (right), with different colors representing different range of reactivity scores. **h** Covariation structure of a specific napRNA, hsa-napRNA-1852 (chr3:193,155,217-193,155,403: -) and hsa-napRNA-2666 (chrX: 20194567-20194942: -). The coevolving base sequences are annotated with a green background pattern. **i** The secondary structure model and SHAPE-MaP reactivity score for each base of hsa-napRNA-1852 and hsa-napRNA-2666, with different colors representing different range of reactivity scores.

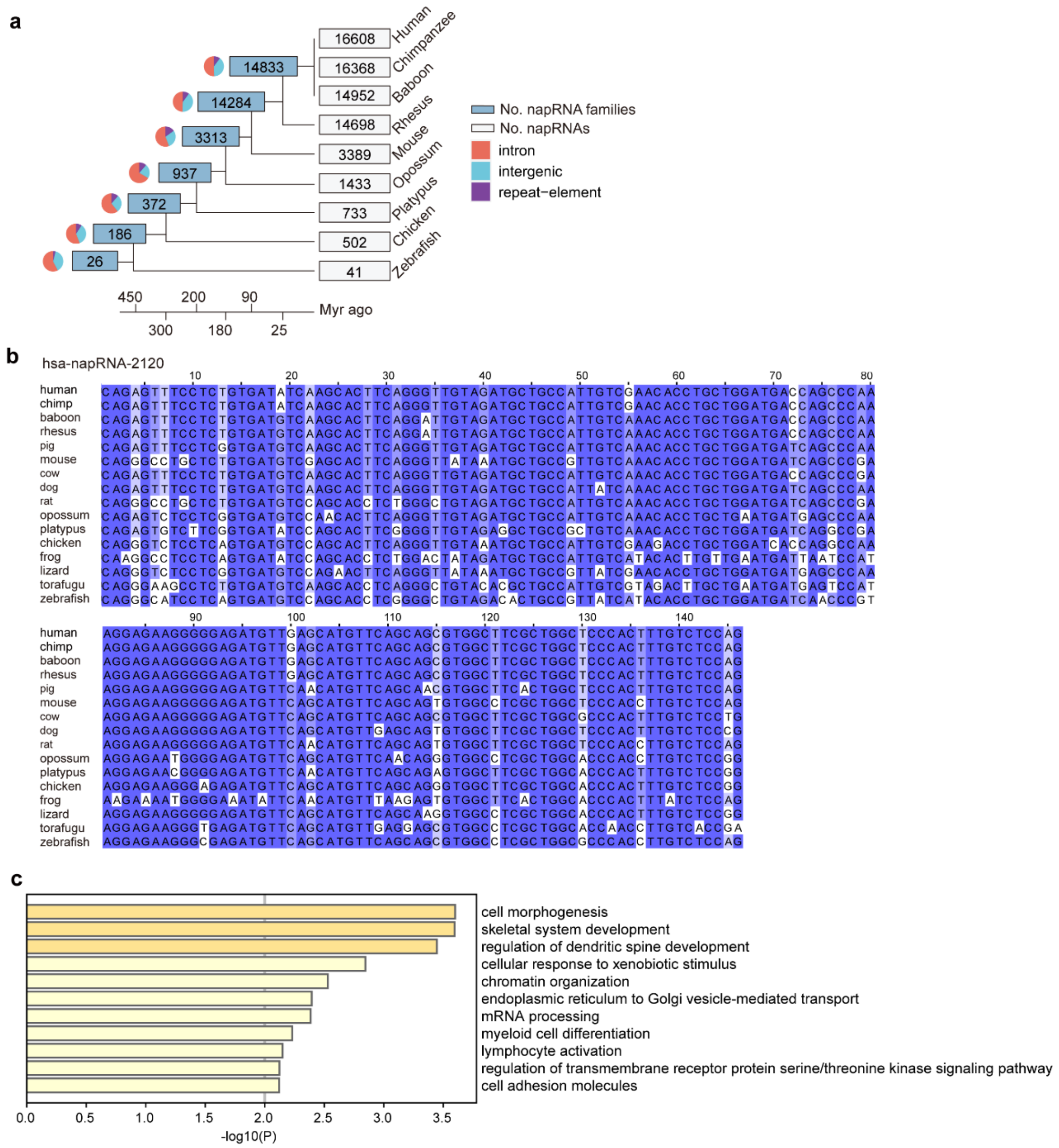


**Supplementary Figure 4. Evolutionary conservation of napRNAs. a** Simplified phylogenetic tree of napRNAs by species. The tree tips indicate the number of napRNAs in each species. The pie plots indicate the genomic distribution of napRNAs with varying degrees of conservation. **b** Sequence alignment for conservation analysis of hsa-napRNA-2120 (https://rnasysu.com/napSeq/structure_browse.php?organism=human&assembly=hg38&protein=&factor_type=napRNA&sample_id=hsa-napRNA-2120). **c** Top enriched Gene Ontology (GO) enriched with the host genes of the napRNAs originated more than 300 Myr ago.

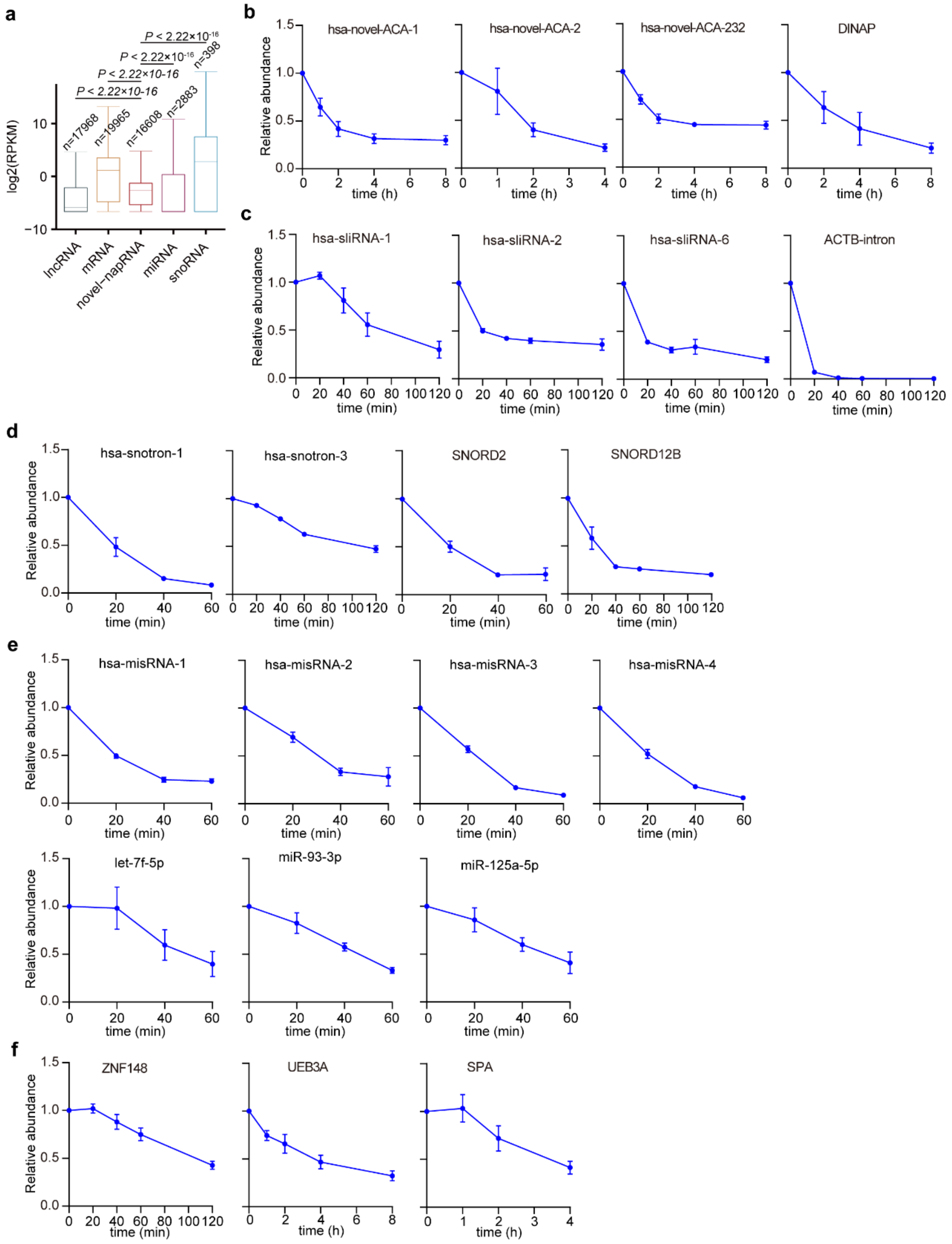


**Supplementary Figure 5. napRNAs are stable rather than byproducts of RNA degradation. a** The steady-state level of napRNAs and other functional RNA species, e.g. mRNA, lncRNA, miRNA and snoRNA, using total RNA-seq data from the GSE88089 dataset and small RNA-seq from ENCSR000CRX dataset. *P* values were calculated by two-sided Mann-Whitney-Wilcoxon test. Each boxplot shows the minima, maxima, centre, bounds of box, whiskers, first and third percentile. **b-f** The RNA half-

life assay of napRNAs in HepG2 cell by using 5µg/ml ActD treatment**.** **b** The RNA half-life of CD-napRNA and ACA-napRNA; **c** The RNA half-life of sliRNA and the ACTB intron which is rapid degraded; **d** The RNA half-life of snotrons and snoRNAs; **e** The RNA half-life of misRNA and miRNA; **f** the The RNA half-life of mRNA and sno-end lncRNA. Source data are provided as a Source Data file.

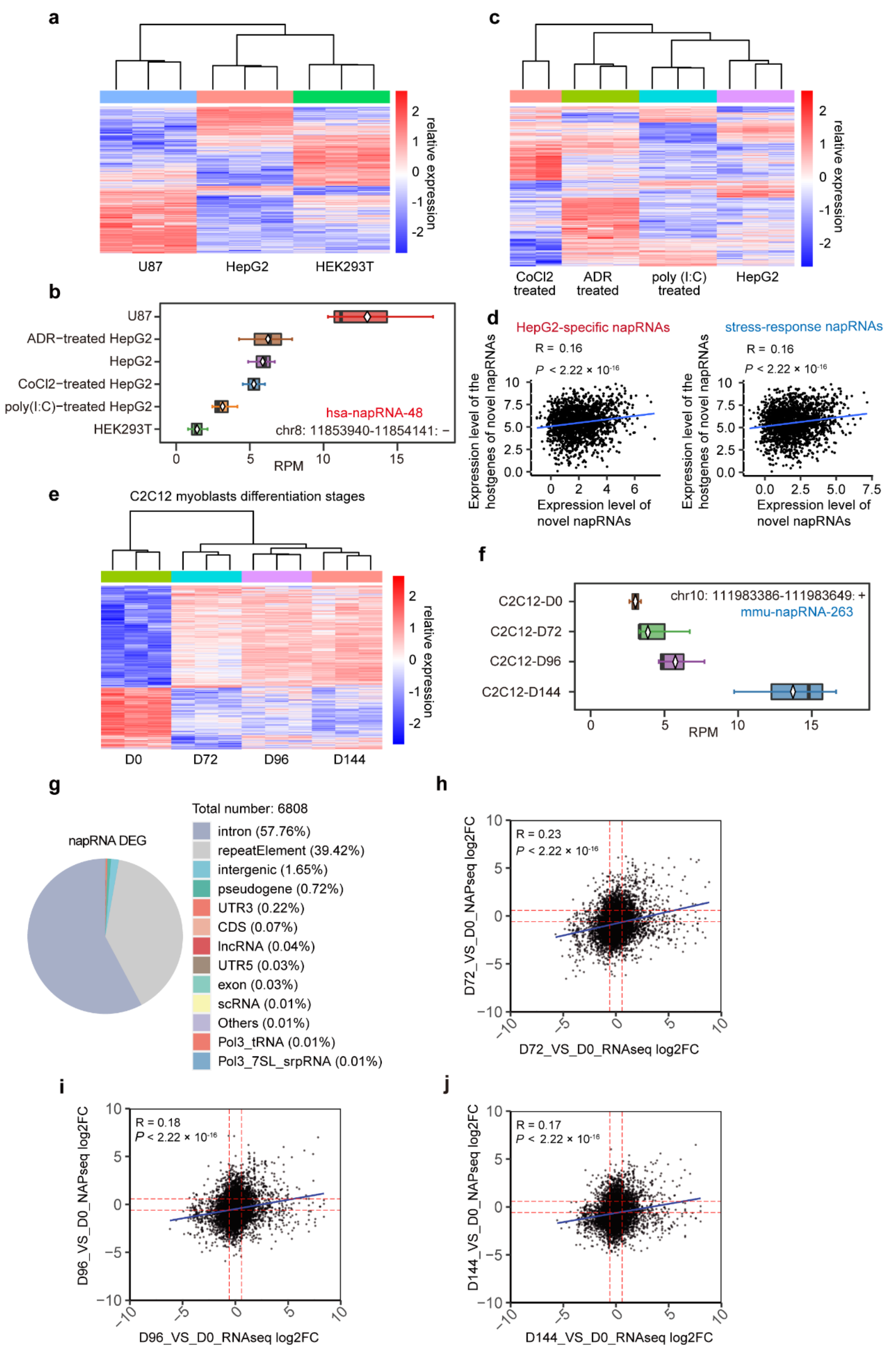


**Supplementary Figure 6. Structured ncRNAs with dynamic expression profiles. a** The heatmap showing the differentially expressed napRNAs in three human cell lines.

**b** Box plot showing the differential expression (3 biological replicates) of hsa-napRNA-48 (chr8:11,853,940-11,854,141: -), which was highly expressed in U87 cells compared to the other cell lines. RPM, reads per million. **c** The heatmap showing the differentially expressed napRNAs during different cellular stress responses. **d** Scatterplot show the correlation between reads (RPM, reads per million) obtained by the NAP-seq (napRNA regions) and RNA-seq (host gene regions) in human. **e** The heatmap showing the differentially expressed napRNAs at different myoblast differentiation stages. Each row in **a**, **c** and **e** represents a differentially expressed napRNA, and each column represents a sample with 3 biological replicates, except for $CoCl_2$ treatment, which had 2 biological replicates. The colour, ranging from blue to red, represents the relative expression values from low to high, respectively. **f** Box plot showing the differential expression (3 biological replicates) of mmu-napRNA-263 (chr10:111,983,386-111,983,649: +), which was differentially expressed during myoblast differentiation. **g** Genomic distribution of differentially expressed napRNAs in mouse C2C12 cells during developmental stages. **h-j** Scatterplot show the correlation of differential gene changes between NAP-seq (napRNAs) and RNA-seq (host mRNAs of napRNAs). *P* values in **d** and **h-j** were calculated by two-sided Pearson's correlation test. D72 represents for C2C12 cells induced to differentiate for 72h; D96 represents for C2C12 cells induced to differentiate for 96h; D144 represents for C2C12 cells induced to differentiate for 144h.

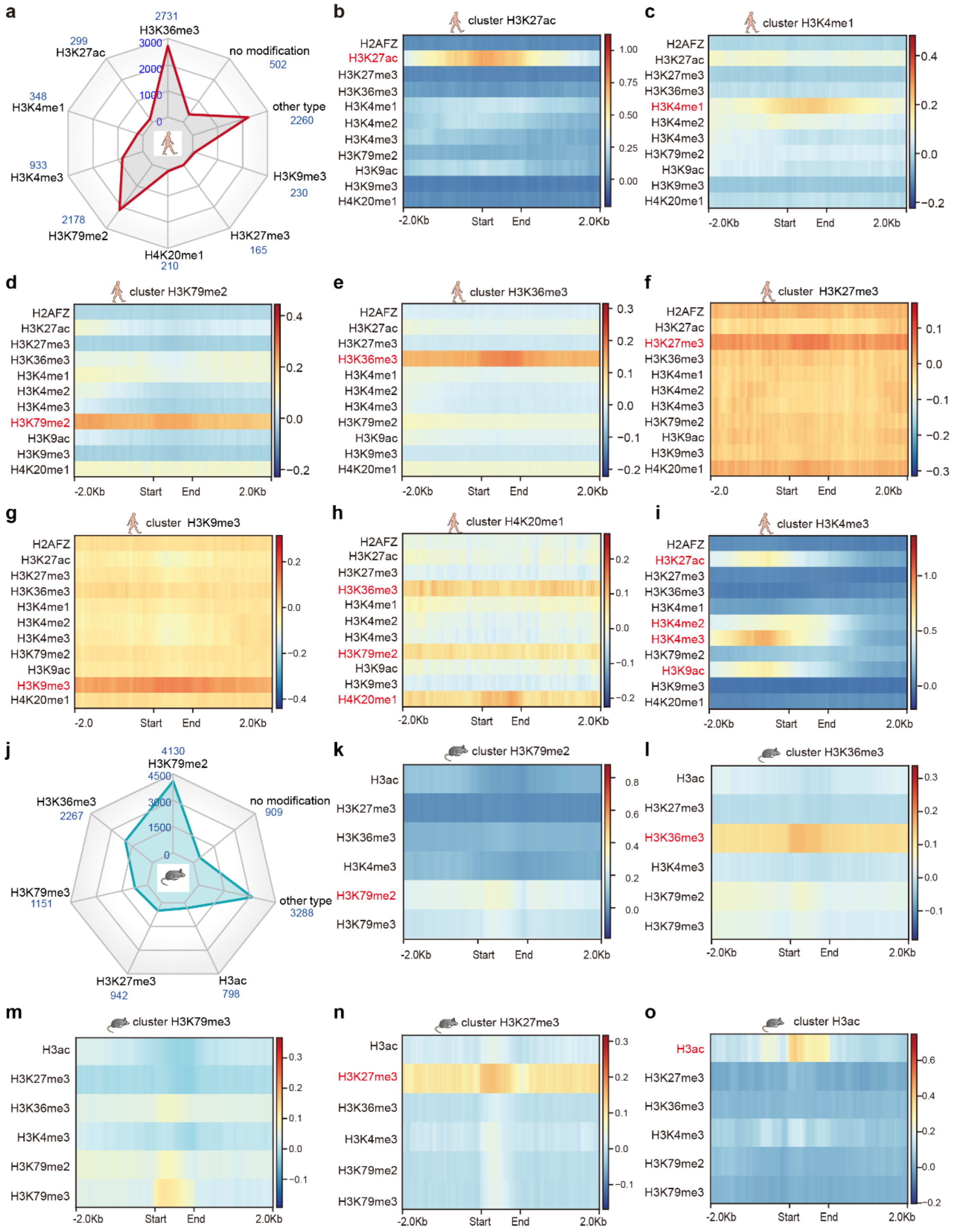


**Supplementary Figure 7. Structured ncRNAs with histone modifications. a** Histone modification status in previously unidentified napRNAs in humans. Radar plots showing the kinds of napRNA clusters and the corresponding numbers. **b-i** Diverse types of histone modifications are abundant in previously unidentified napRNAs in humans. The x-axis shows the regions of napRNA bodies and the regions up to 2 kb upstream and downstream. The clusters H3K27ac (**b**), H3K4me1 (**c**), H3K79me2 (**d**),

H3K36me3 (**e**), H3K27me3 (**f**) and H3K9me3 (**g**) represent the kinds of histone modifications that were dominant, while clusters H4K20me1 (**h**) and H3K4me3 (**i**) represent various histone modifications that worked in synergy. **j** Histone modification patterns of previously unidentified napRNAs in mice. Radar plots showing various kinds of napRNA clusters and the corresponding numbers. **k-o** Clusters of histone modifications within napRNAs in mice.

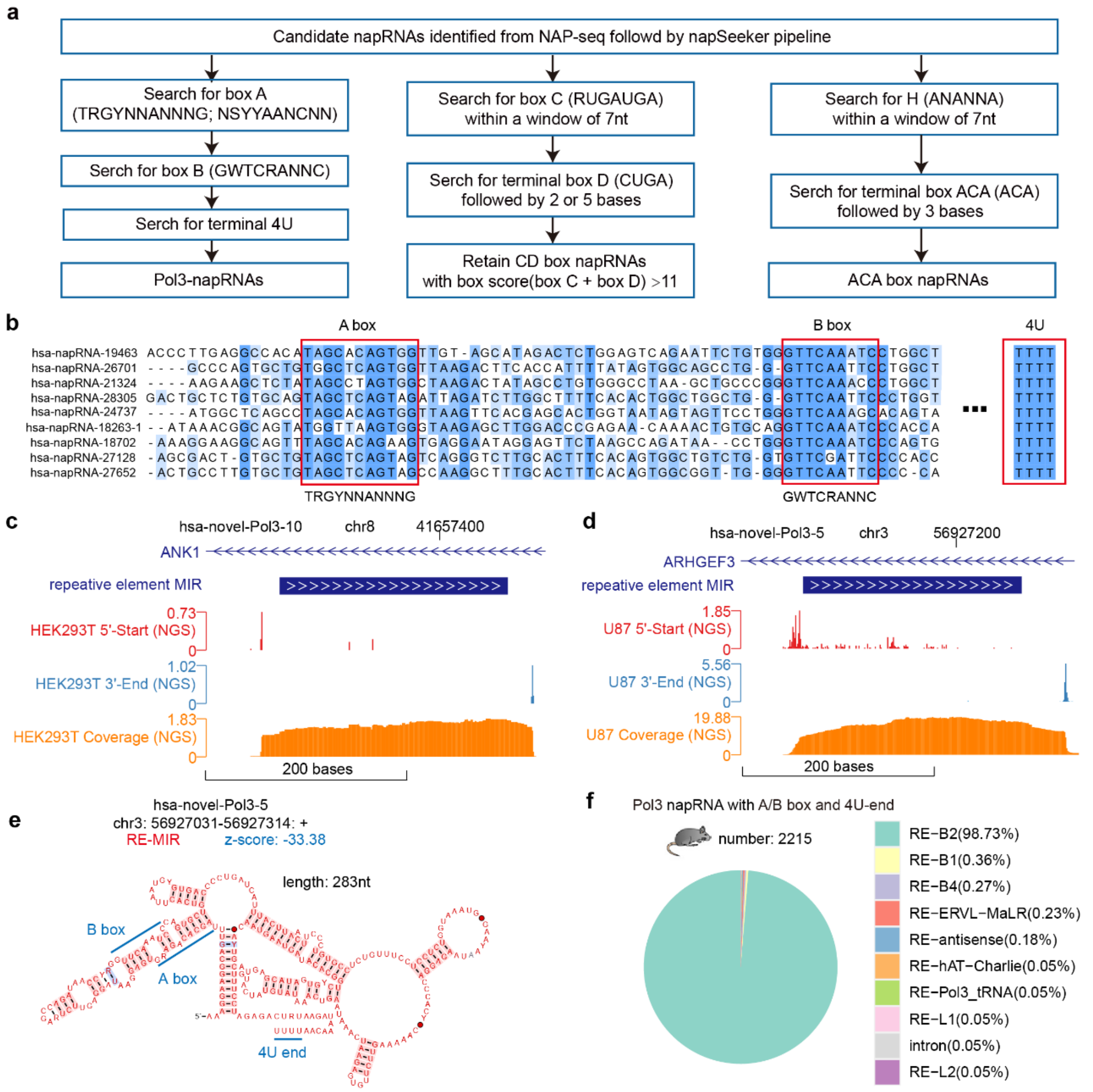


**Supplementary Figure 8. Computational pipeline for discovering several subgroups of ncRNAs and characteristics of Pol3-napRNAs. a** An overview of the computational pipeline developed for the identification of a previously unknown subgroup of ncRNAs. **b** Multiple sequence alignment of the previously unidentified Pol3-napRNAs. The relatively conserved A/B box sequences and 4U tails are highlighted in red boxes. **c and d** Genome Browser view of 5'-start, 3'-end and coverage signals (RPM, reads per million) in an extended region of Pol3-napRNAs at single-nucleotide resolution. **e** The highly stable secondary structure of hsa-novel-Pol3-5. A negative z score indicated that a sequence was more stable than expected by chance. **f** Distribution of candidate mouse Pol3-napRNAs in annotated gene types. Only the napRNAs located within intergenic, intronic, and repetitive elements were retained and

are shown in the figure. RE, repetitive elements.

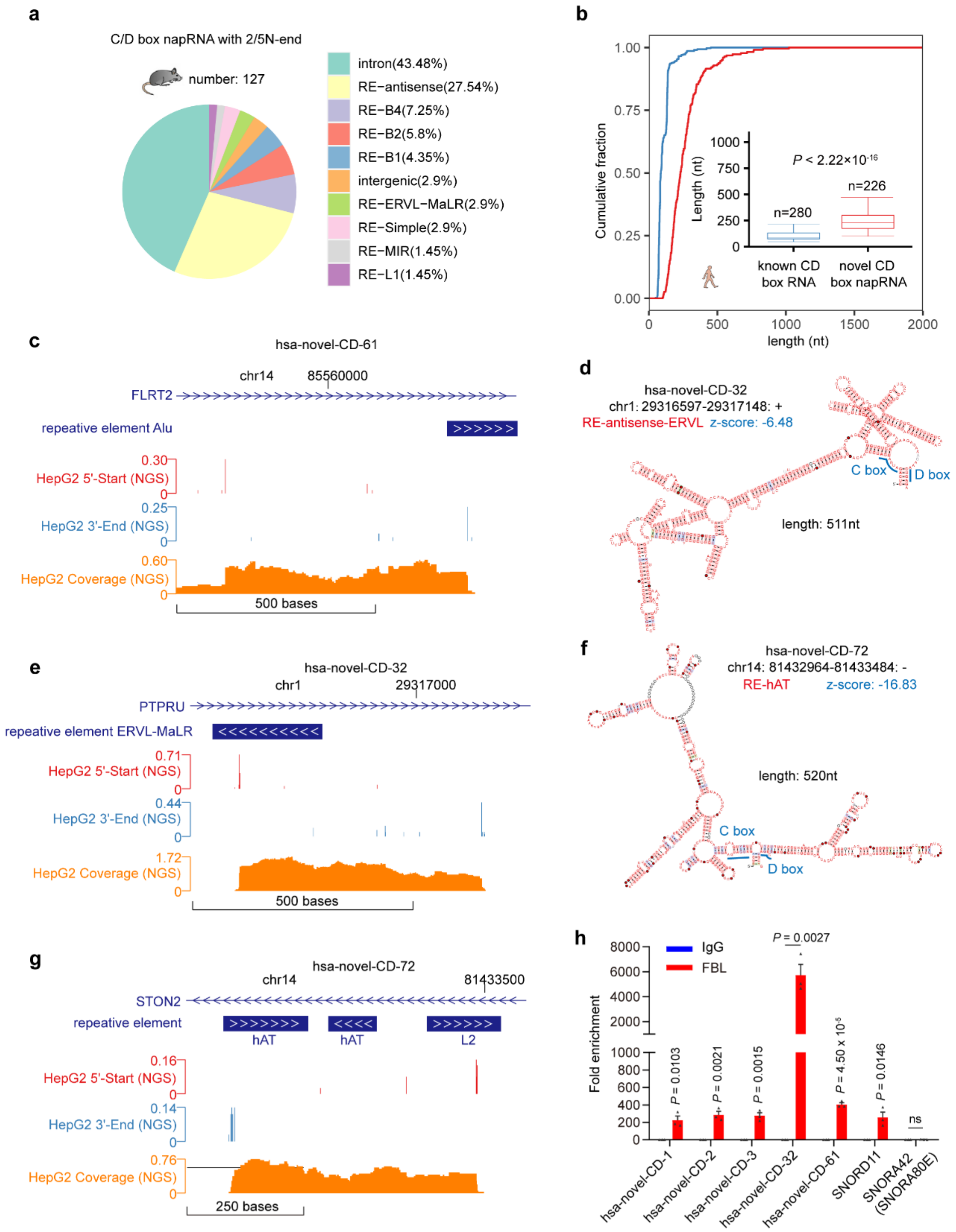


**Supplementary Figure 9. Characteristics of C/D box napRNAs. a** Distribution of candidate mouse C/D-napRNAs in annotated gene types. Only the napRNAs located within intergenic, intronic, and repetitive elements were retained and are shown in the figure. **b** Cumulative curves and box plots showing the lengths of previously unidentified C/D-napRNAs and known C/D box snoRNAs (from snoRNABase). *P*

value was calculated by two-sided Mann-Whitney-Wilcoxon test. Each boxplot shows the minima, maxima, centre, bounds of box, whiskers, first and third percentile. **c-g** Genome Browser view (**c, e and g)** and RNA secondary structure (**d and f**) of C/D-napRNAs. A negative z score indicated that a sequence was more stable than expected by chance. **h** The interaction between FBL protein and snoRNAs identified by NAP-seq were confirmed by RIP-qPCR in HepG2 cells. The values are mean ± SEM of 3 independent experiments. Two-sided t-test. ns, not significant. Source data are provided as a Source Data file.

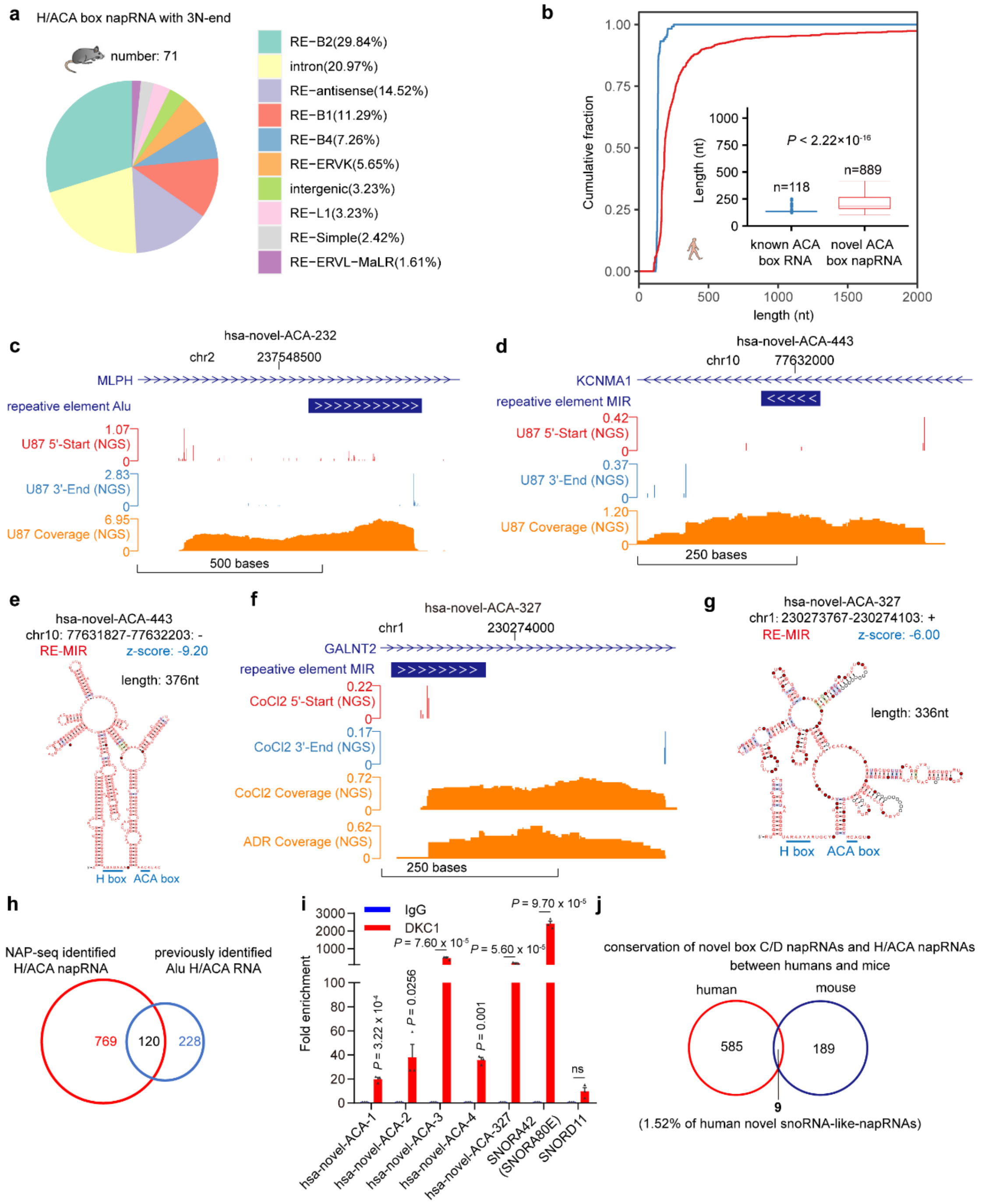


**Supplementary Figure 10. Characteristics of H/ACA box napRNAs. a** Distribution of candidate mouse H/ACA-napRNAs in annotated gene types. Only the napRNAs located within intergenic, intronic, and repetitive elements were retained and are shown in the figure. **b** Cumulative curves and box plots showing the lengths of previously unidentified H/ACA-napRNAs and known H/ACA box snoRNAs (from snoRNABase). *P* value was calculated by two-sided Mann-Whitney-Wilcoxon test. Each boxplot shows the minima, maxima, centre, bounds of box, whiskers, first and third percentile.

**c-g** Genome Browser view (**c, d and f**) and secondary structure (**e and g**) of H/ACA-napRNAs. A negative z score indicated that a sequence was more stable than expected by chance. **h** The intersection of H/ACA-napRNAs identified by NAP-seq and the previously identified Alu-H/ACA snoRNAs. **i** The interaction between DKC1 protein and snoRNAs identified by NAP-seq were confirmed by RIP-qPCR in HepG2 cells. **j** Intersection of snoRNA-like napRNAs identified from humans and mice. For conservation analysis, 521 Alu-ACA napRNAs identified by NAP-seq were excluded from the 1115 snoRNA-like napRNAs in human. The values in **i** are mean ± SEM of 3 independent experiments. Two-sided t-test.ns, not significant. Source data are provided as a Source Data file.

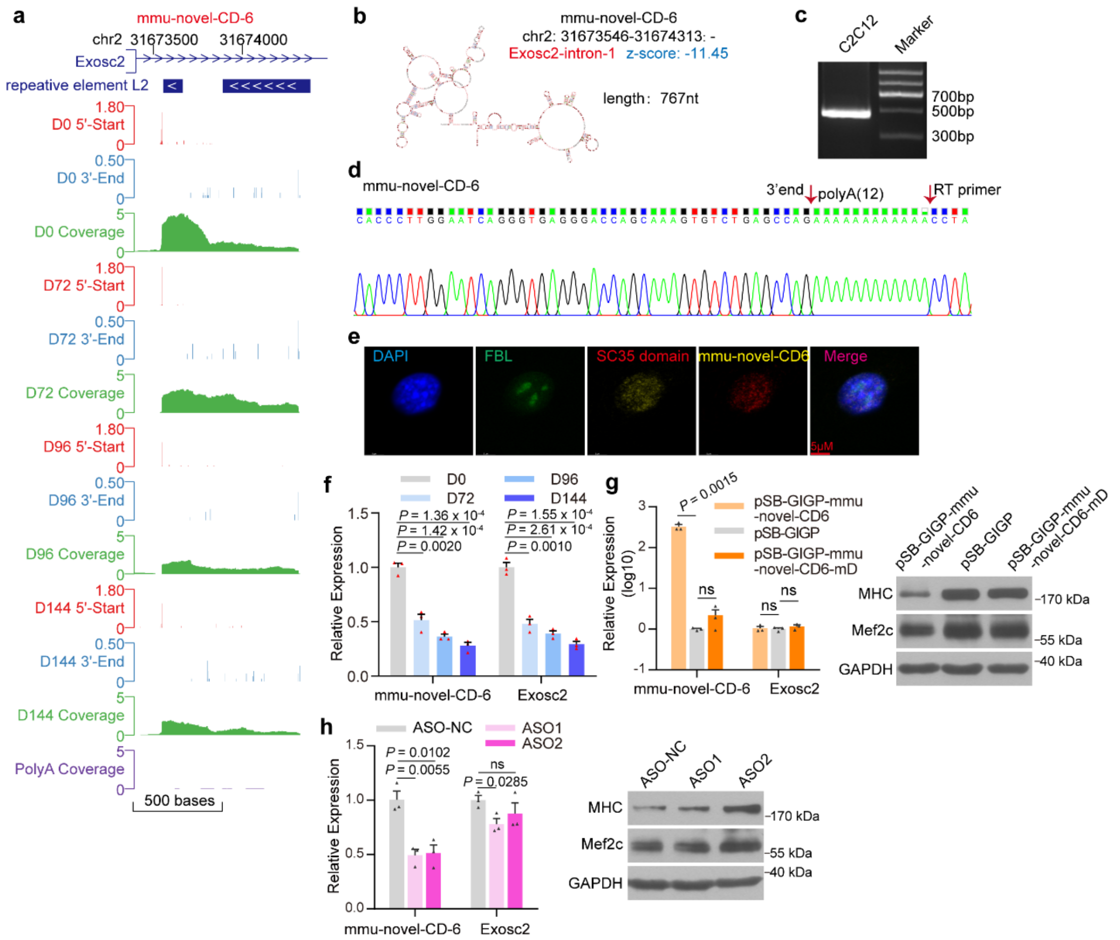


**Supplementary Figure 11. mmu-novel-CD-6, derived from a repetitive element, inhibited C2C12 myoblast differentiation. a** The dynamic expression of mmu-novel-CD-6 during C2C12 myoblast differentiation. Genome Browser view of 5'-start, 3'-end and coverage signals (RPM, reads per million) in an extended region of mmu-novel-CD-6. **b** The highly stable secondary structure of mmu-novel-CD-6. The detailed information and the z score calculated by RNAz are shown. **c and d** The 3'-end site in mmu-novel-CD-6 was confirmed by poly(T) RT–PCR (**c**) coupled with Sanger sequencing (**d**). **e** Subcellular localization of mmu-novel-CD-6 (yellow), as assessed by smiFiSH. Nuclear DNA was stained with DAPI (blue). Simultaneously, FBL protein (green) and SC-35 domains (red) was stained by immunofluorescence (IF). All images are representative of two biological replicates. **f** qPCR was used to assess the RNA expression of mmu-novel-CD-6 and its host gene-Exosc2 when C2C12 myoblasts were induced to differentiate for 0 h (D0), 72 h (D72), 96 h (D96) and 144 h (D144). **g** The expression levels of mmu-novel-CD-6 and Exosc2 were determined by qPCR in mmu-

novel-CD-6-overexpressing C2C12 cells and mmu-novel-CD-6 mutant with D box mutation-overexpressing cells (mmu-novel-CD6-mD) at 48 h (D48). Western blotting analysis showed the level of MHC and Mef2c compared with that in control C2C12 cells at 48 h (D48). **h** The expression levels of mmu-novel-CD-6 and Exosc2 were determined by qPCR in mmu-novel-CD-6 knockdown C2C12 cells at 48 h (D48). Western blotting analysis showed the level of MHC and Mef2c compared with that in control C2C12 cells at 48 h (D48). The values in **f, g** and **h** are mean ± SEM of 3 independent experiments. Two-sided t-test. Source data are provided as a Source Data file..

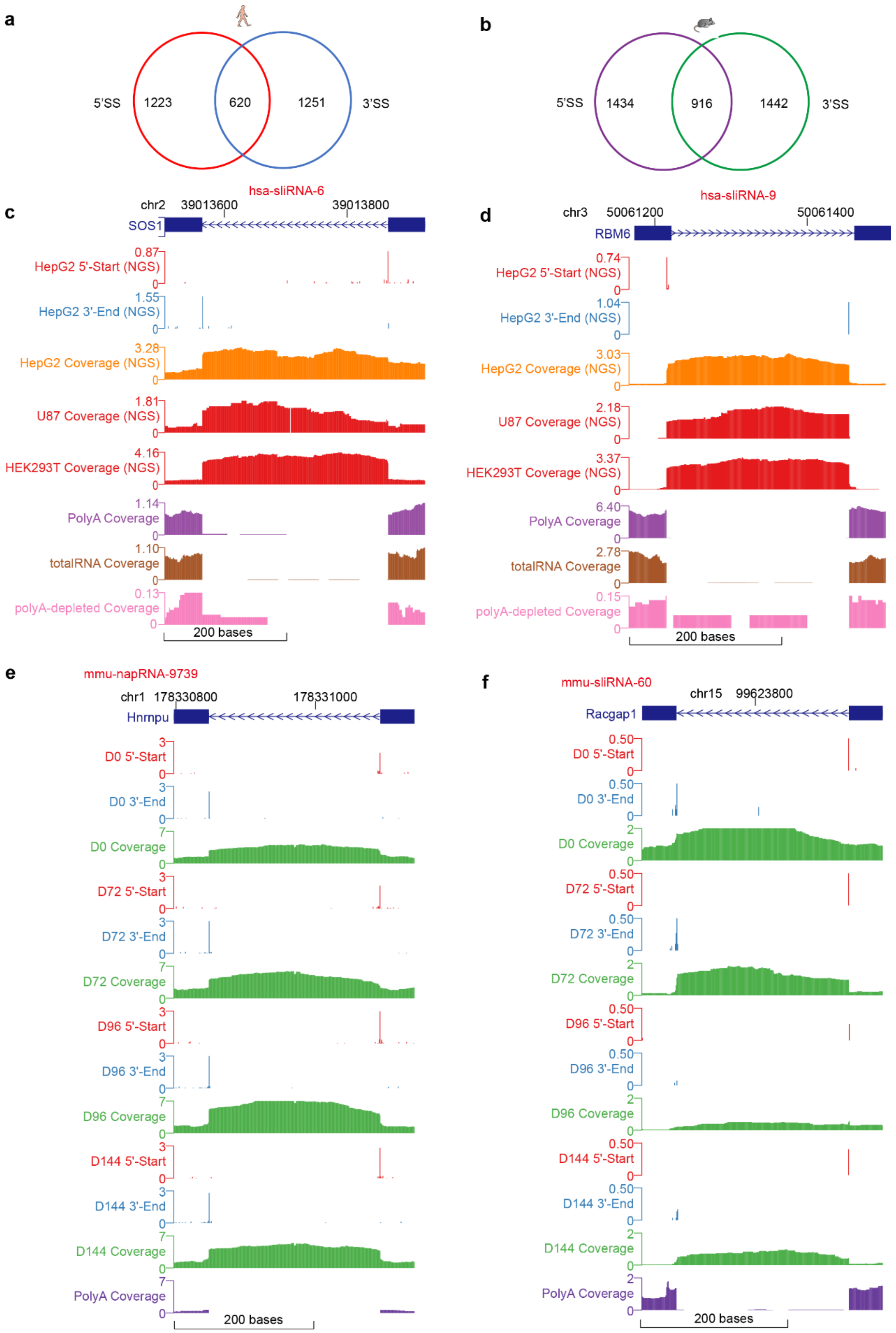


**Supplementary Figure 12. Dynamic expression of sliRNAs. a and b** The number of overlapping napRNAs harbouring both a 5'-SS and 3'-SS in humans (**a**) and mice (**b**); these napRNAs were named sliRNAs. **c and d** The expression of sliRNAs in human

cells. Genome Browser view of both terminal sites and read coverage (RPM) in an extended region of sliRNAs in humans. **e and f** The dynamic expression of sliRNAs during myoblast differentiation. Genome Browser view of both terminal sites and read coverage (RPM, reads per million) in an extended region of sliRNAs during myoblast differentiation.

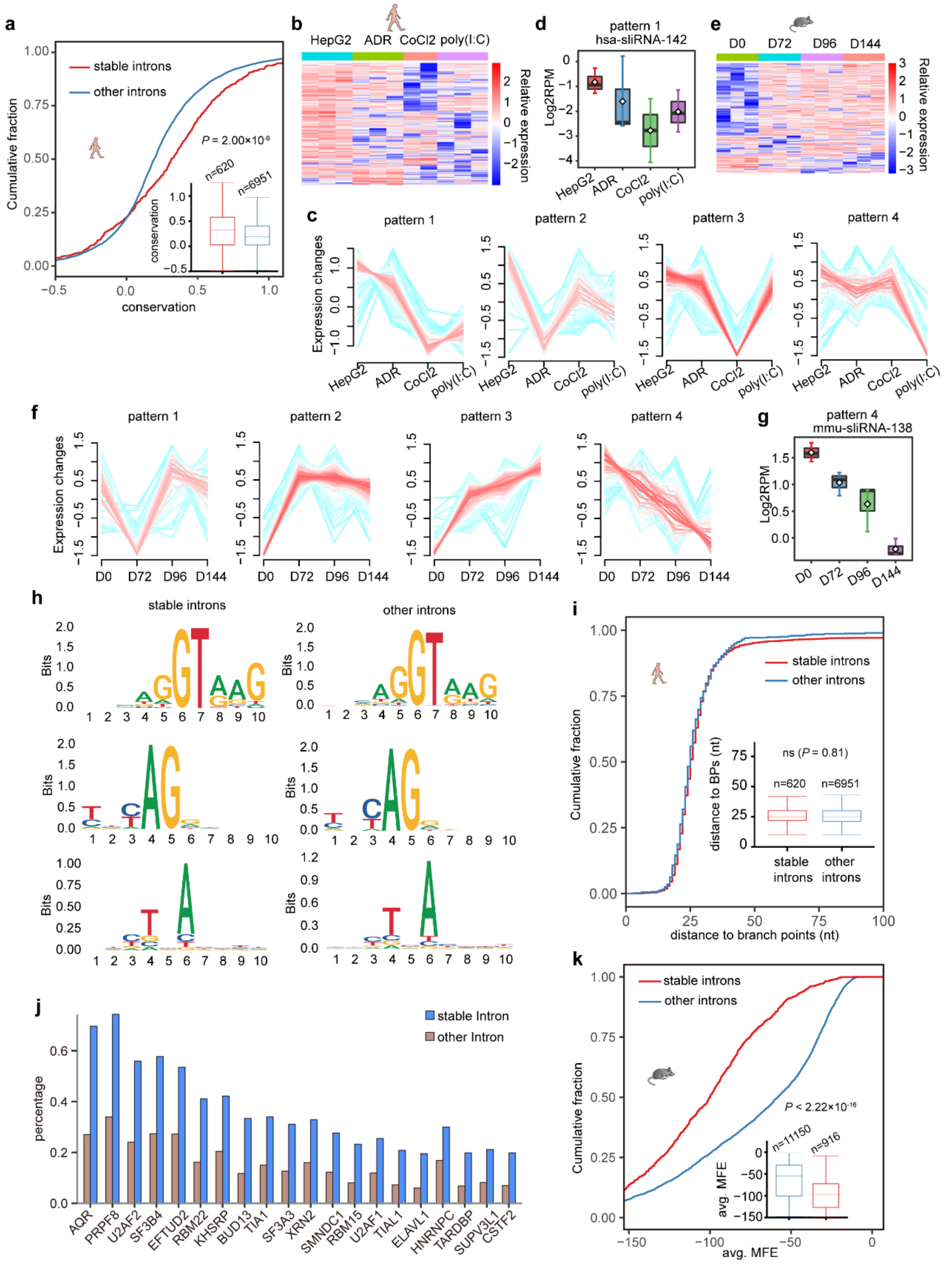


**Supplementary Figure 13. Genomic features of sliRNAs. a** Cumulative curves and box plots showing the conservation scores of sliRNAs (stable introns) and other introns in humans. **b** The heatmap showing the differentially expressed sliRNAs in HepG2 cells under control and three stress-response treatment conditions. Each row represents a differentially expressed sliRNA, and each column represents a treatment type. The colour, ranging from blue to red, represents the relative expression values from low to

high, respectively. **c** Stress-response expression profiles of sliRNAs in HepG2 cells. The c-means fuzzy clustering method identified four different stress-response patterns. The x-axis shows the four treatments in HepG2 cells, while the y-axis shows the log2-transformed, normalized intensity ratios in each condition. **d** Expression values of representative genes of pattern 1 in **c**. Box plot showing the log2 RPM values in HepG2 cells under four conditions. Each box shows the first quartile, median, and third quartile. **e** The heatmap showing the differentially expressed sliRNAs in mouse C2C12 cells at four developmental stages. **f** Temporal expression profiles of sliRNAs in C2C12 cells. The c-means fuzzy clustering method identified four different temporal patterns. The x-axis shows the four developmental stages, while the y-axis shows the log2-transformed, normalized intensity ratios in each stage. **g** Expression values of representative genes of pattern 4 in **f**. Box plot showing the log2RPM (RPM, reads per million) values in C2C12 cells at four stages. Each box shows the first quartile, median, and third quartile. **h** The consensus sequences of splicing signals, including the 5' splice sites (top), 3' splice sites (middle) and branch points (bottom) of sliRNAs and other introns. The x-axis shows the relative base position, and the y-axis shows the base identity. **i** Cumulative curves and box plots showing the distance from intron 3' splice sites to branch points (A) in human sliRNAs. Each box shows the first quartile, median, and third quartile. ns, not significant. **j** Comparison of RBP binding to sliRNAs or other introns. The RBP-binding percentages of sliRNAs and other introns are shown. The x-axis shows the RBPs (top 20), and the y-axis shows the percentage of introns that can be bound by the RBP among all introns. **k** Cumulative curves and box plots showing the MFE values of sliRNAs and other introns with a length $\leq$500 nt in mice. Each boxplot in **a**, **i** and **k** shows the minima, maxima, centre, bounds of box, whiskers, first and third percentile. *P* values in **a**, **i** and **k** were calculated by two-sided Mann-Whitney-Wilcoxon test.

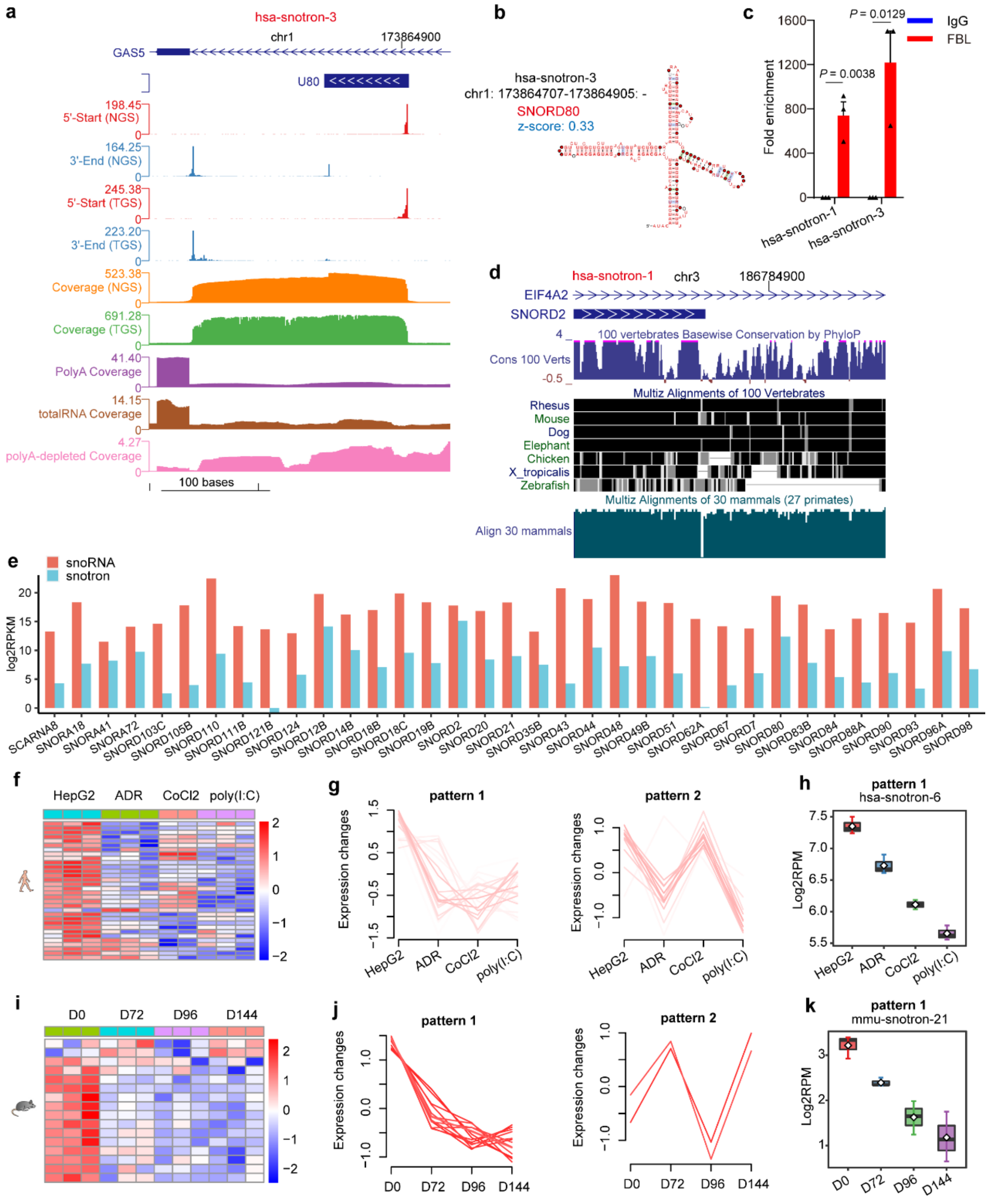


**Supplementary Figure 14. Evolutionary conservation and characteristics of snotrons. a** The expression profile of snotrons in humans. Genome Browser view of 5'-start, 3'-end and coverage signals (RPM, reads per million) in an extended region of the snotron hsa-snotron-3 (chr1:173,864,707-173,864,905). **b** The secondary structure of snotron hsa-snotron-3. **c** The interaction between the FBL and snotrons containing C/D box snoRNA sequences was confirmed by RIP-qPCR in HepG2 cells. The values are mean ± SEM of 3 independent experiments. Two-sided t-test. Source data are provided as a Source Data file. **d** Evolutionary conservation analysis of the snotron hsa-

snotron-1 (chr3:186,784,797-186,784,960) is shown by the conservation score (PhyloP) and multiple sequence alignment in 100 vertebrate species. **e** Comparion of the snoRNA and snotron expression. The expression of snoRNAs were calculated by PEN-seq in HepG2 (GSE160887 [https://www.ncbi.nlm.nih.gov/geo/query/acc.cgi?acc=GSE160887]). Snotron expression was compared to snoRNAs by removing the batch effect, correcting the library depth, and normalizing the gene length. **f** The heatmap showing the differentially expressed snotrons in HepG2 cells under control and three stress-response treatment conditions. Each row represents a differentially expressed snotron, and each column represents a treatment type. The colour, ranging from blue to red, represents the relative expression values from low to high, respectively. **g** Stress-response expression profiles of snotrons in HepG2 cells. The c-means fuzzy clustering method identified two different stress-response patterns. The x-axis shows the four stress conditions in HepG2 cells, while the y-axis shows the log2-transformed, normalized intensity ratios under each condition. **h** Expression values of representative genes of pattern 1 in **g**. Box plot showing the log2RPM (RPM, reads per million) values in HepG2 cells under four conditions. Each box shows the first quartile, median, and third quartile. **i** The heatmap showing the differentially expressed snotrons in mouse C2C12 cells at four developmental stages. **j** Temporal expression profiles of snotrons in C2C12 cells. The c-means fuzzy clustering method identified two different temporal patterns. The x-axis shows the four developmental stages, while the y-axis shows the log2-transformed, normalized intensity ratios in each stage. **k** Expression values of representative genes of pattern 1 in **j**. Box plot showing the log2RPM (RPM, reads per million) values in C2C12 cells at four stages. Each box shows the first quartile, median, and third quartile.

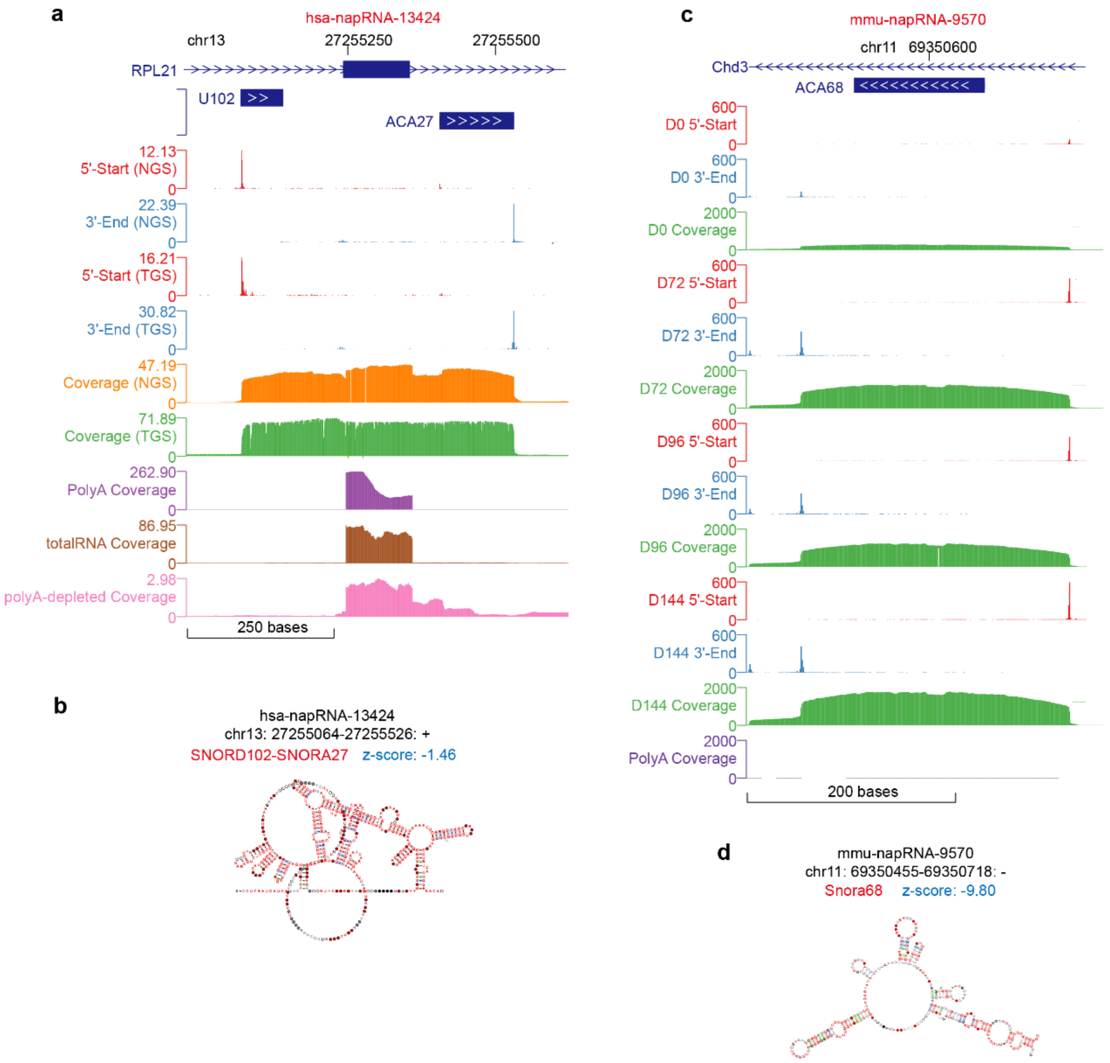


**Supplementary Figure 15. Previously undiscovered sno-end lncRNAs identified by NAP-seq. a** The expression profiles of previously undiscovered sno-end lncRNA in humans. Genome Browser view of 5'-start, 3'-end and coverage signals (RPM, reads per million) in an extended region of sno-end lncRNA. **b** The secondary structure of previously unidentified sno-end lncRNA. **c** The dynamic expression profile of reannotated ACA68 (napACA68, mmu-napRNA-9570), with a long length, during myoblast differentiation. Genome Browser view of 5'-start, 3'-end and coverage signals (RPM, reads per million) in an extended region of napACA68**. d** The secondary structure of the napACA68.

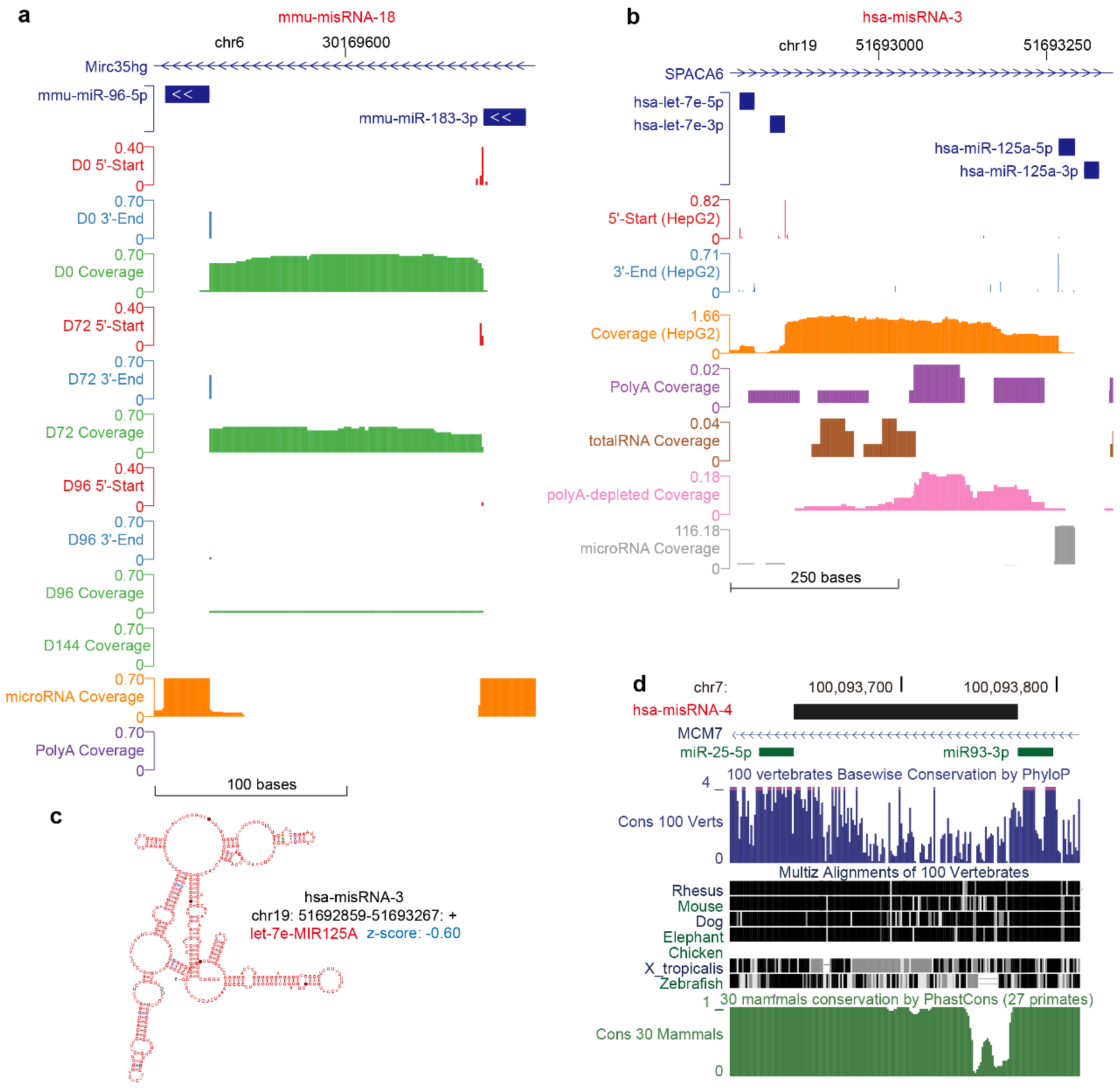


**Supplementary Figure 16. Examples of misRNAs. a** Genome Browser view of the full-length misRNAs (RPM, reads per million) in an extended region of misRNA mmu-misRNA-18. **b** Genome Browser view of coverage signals (RPM, reads per million) in an extended region of misRNA hsa-misRNA-3**. c** The secondary structure of the misRNA hsa-misRNA-3. **d** Evolutionary conservation analysis of the misRNA hsa-misRNA-4 (chr7:100,093,630-100,093,775: -) by the conservation score (PhyloP) and multiple sequence alignment in 100 vertebrate species.

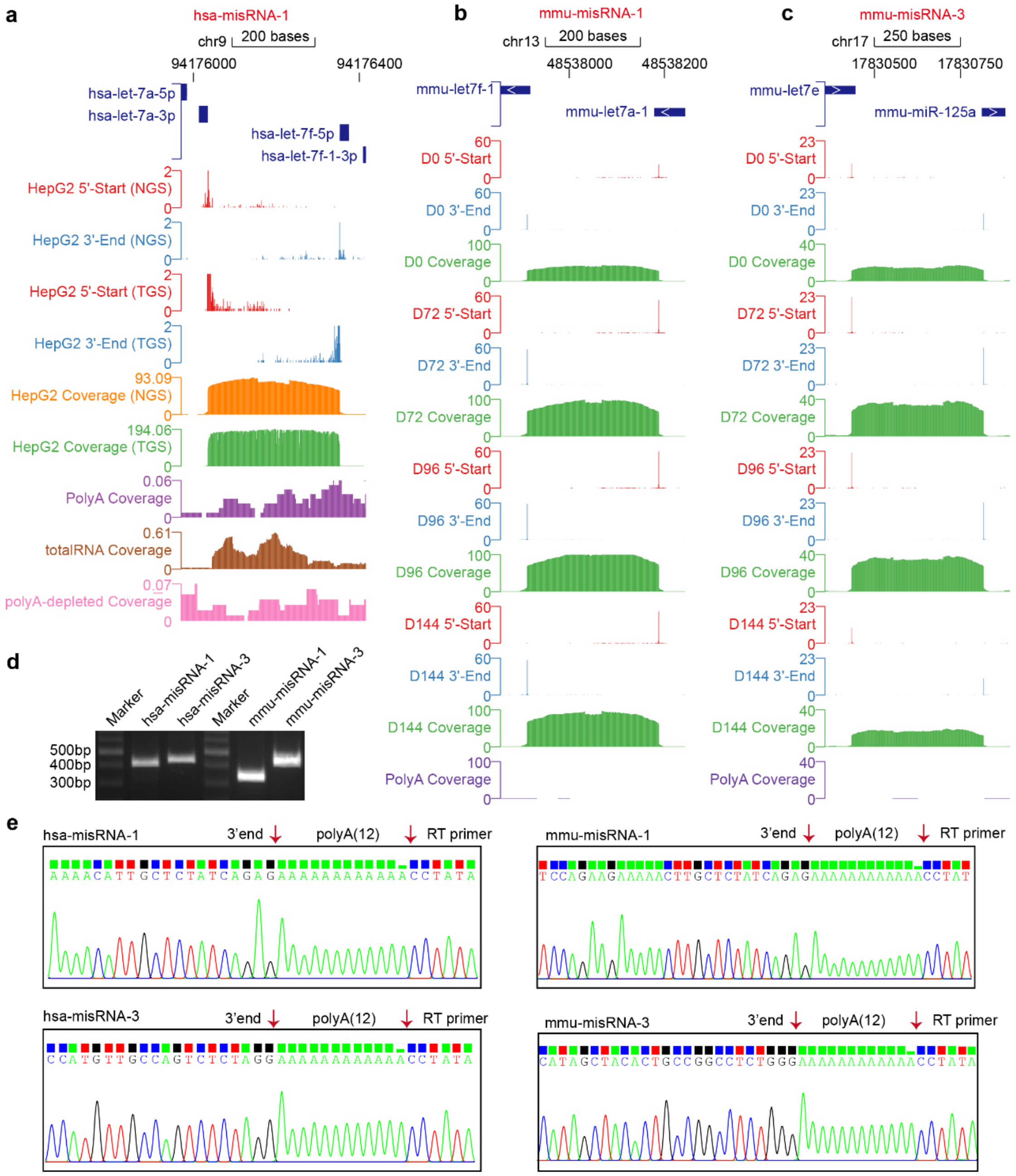


**Supplementary Figure 17. Verification of homologous misRNAs in humans and mice by poly(T) RT–PCR coupled with Sanger sequencing. a-c** The dynamic expression of misRNAs in humans and mice. Genome Browser view of the coverage signals (RPM, reads per million) in an extended region of the misRNAs hsa-misRNA-1 (**a**), mmu-misRNA-1 (**b**) and mmu-misRNA-3 (**c**). **d** The verification of misRNAs by using poly(T) RT–PCR. **e** The validation of misRNAs by Sanger sequencing. The precise 3'-end sites in hsa-misRNA-1, hsa-misRNA-3, mmu-misRNA-1, and mmu-misRNA-3 were identified using Sanger sequencing.

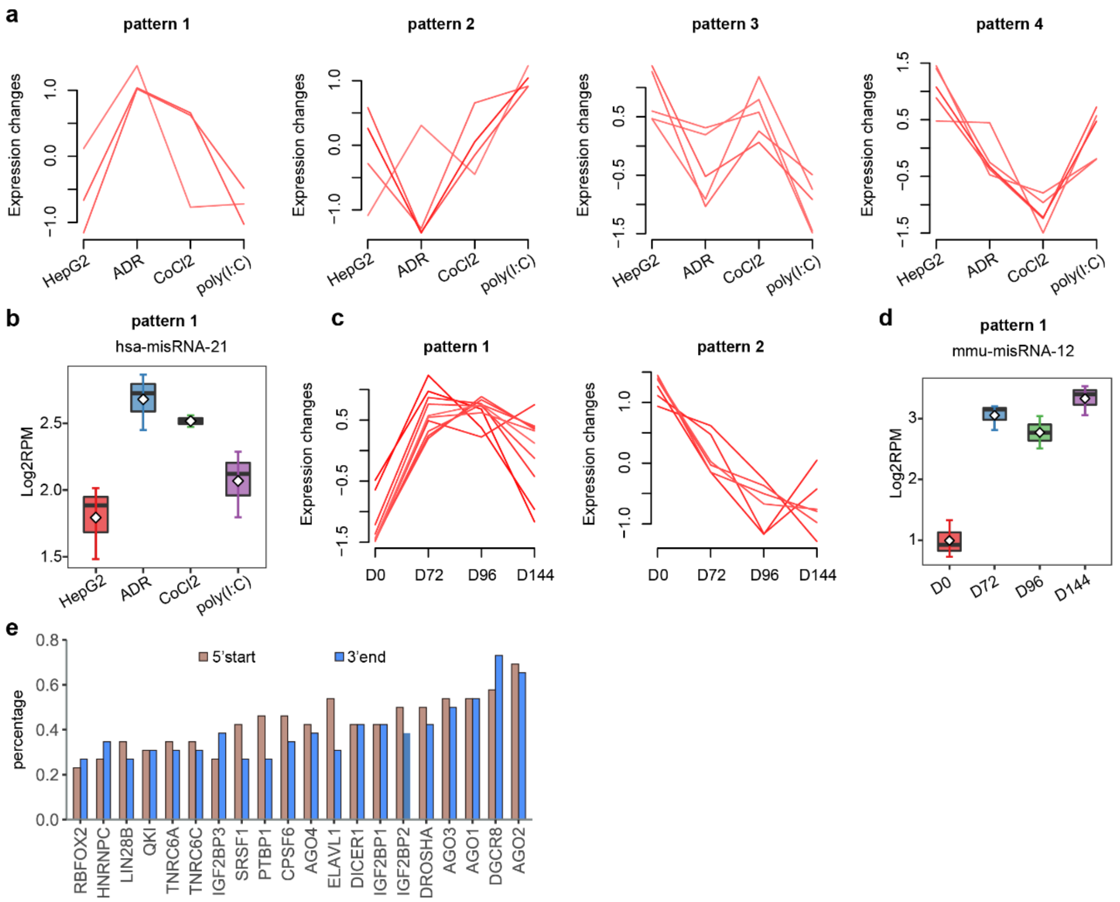


**Supplementary Figure 18. Dynamic expression profiles of misRNAs and illustration of miRNA biogenesis. a** Stress-response expression profiles of misRNAs in HepG2 cells. The c-means fuzzy clustering method identified four different stress-response patterns. The x-axis shows the four stress conditions in HepG2 cells, while the y-axis shows the log2-transformed, normalized intensity ratios under each condition. **b** Expression values of representative genes of pattern 1 in **a**. Box plot showing the log2RPM (RPM, reads per million) values in HepG2 cells under the four conditions. Each box shows the first quartile, median, and third quartile. **c** Temporal expression profiles of misRNAs during myoblast differentiation. The c-means fuzzy clustering method identified two different temporal patterns. The x-axis shows the four developmental stages, while the y-axis shows the log2-transformed, normalized intensity ratios in each stage. **d** Expression values of representative genes of pattern 1 in **c**. Box plot showing the log2RPM (RPM, reads per million) values in C2C12 cells in the four stages. Each box shows the first quartile, median, and third quartile. **e** Comparison of the RBP-binding percentage between misRNA 5'-start and 3'-end

sequences. The x-axis shows the RNA-binding proteins (top 20), and the y-axis shows the percentage of misRNA 5’-start (or 3’-end) sequences that can be bound by a certain RBP.

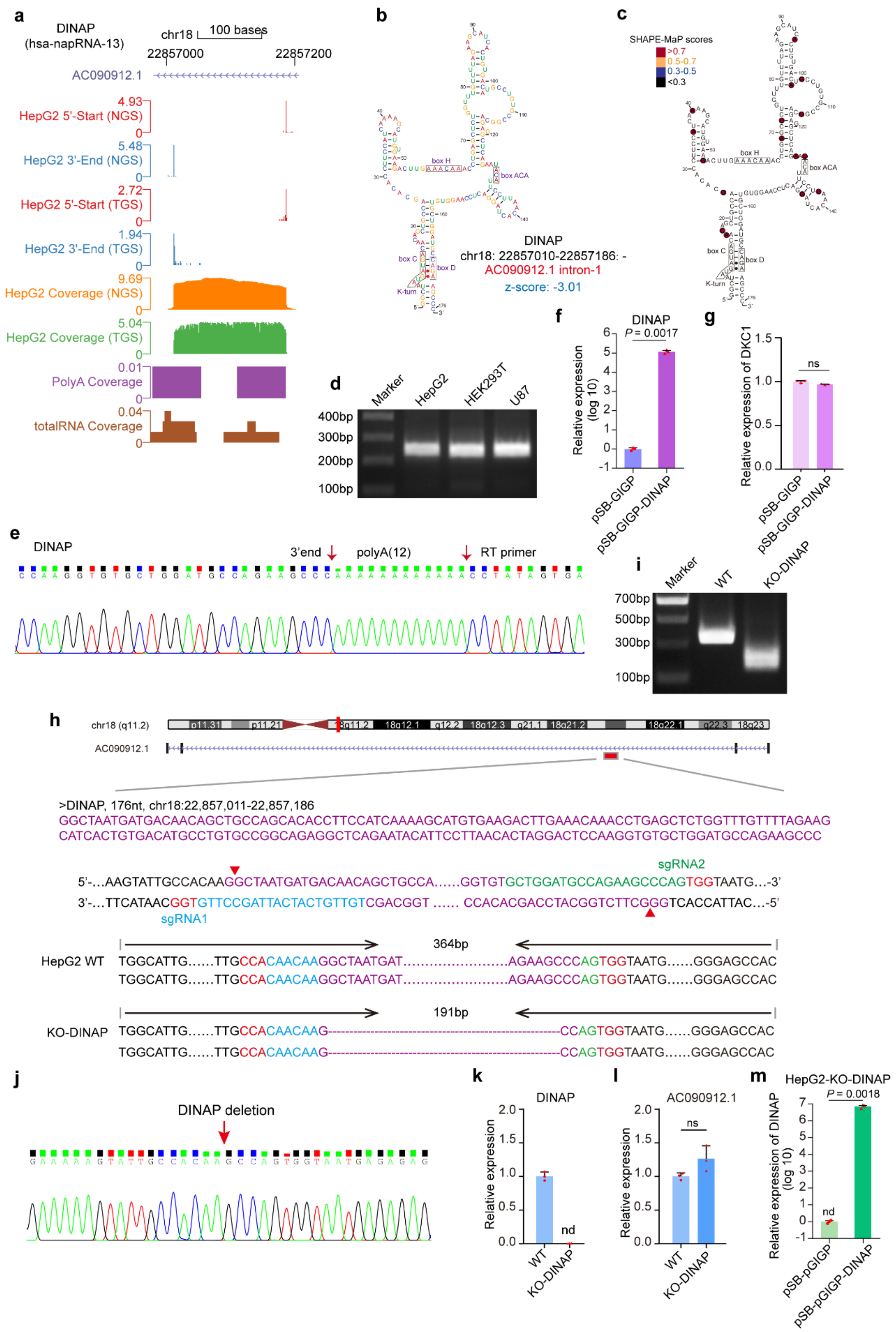


**Supplementary Figure 19. Verification and knockout of DINAP by CRISPR/Cas9.** **a** Genome Browser view of 5'-start, 3'-end and coverage signals (RPM, reads per million) in an extended region of the napRNA DINAP. **b** The highly stable secondary

structure of DINAP. **c** The secondary structure model and NAP-SHAPE-MaP reactivity for each base of DINAP, with different colors representing different range of reactivity scores. **d and e** The 3'-end site in DINAP was verified by poly(T) RT–PCR in three cell lines (HepG2, HEK293T and U87) (**d**) and by Sanger sequencing (**e**). **f** The mRNA expression level of DINAP was assessed by qPCR in DINAP-overexpressing cells generated using recombinant plasmids. **g** The mRNA expression level of DKC1 was determined by qPCR in DINAP-overexpressing HepG2 cells (showing a decrease of approximately 4%). **h** The design of sgRNAs for DINAP KO using the CRISPR/Cas9 system. **i and j** The expression of DINAP in DINAP-KO HepG2 cells was verified by RT–PCR (**i**) and Sanger sequencing (**j**). **k** The expression level of DINAP was measured by qPCR in DINAP-KO cells. **l** The expression level of the DINAP host gene was measured by qPCR in DINAP-KO cells. **m** The expression level of DINAP was determined by qPCR after DINAP expression was rescued in DINAP-KO cells. Nd, not detected; ns, not significant. The values in **f, g, k, l** and **m** are mean ± SEM of 3 independent experiments. Two-sided t-test. Source data are provided as a Source Data file.

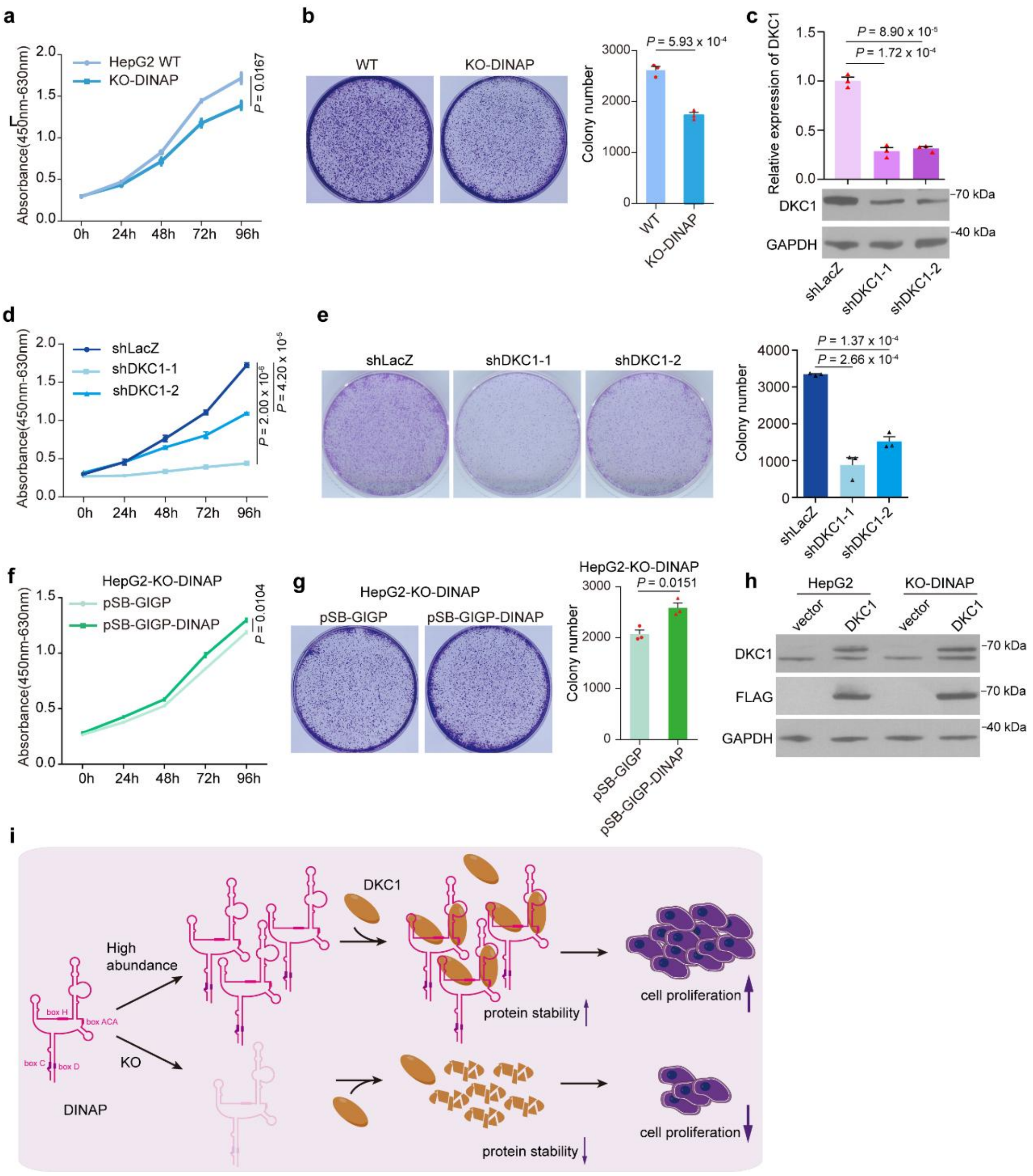


**Supplementary Figure 20. The napRNA DINAP promotes cell proliferation. a** Inhibition of cell proliferation was examined by a CCK-8 assay in DINAP-KO cells and control cells. **b** Effect of DINAP KO on colony formation ability. **c** The expression level of DKC1 was examined by qPCR and western blotting in DKC1-knockdown HepG2 cells. **d** CCK-8 assays showing the suppressive effect of DKC1 on proliferation in DKC1-knockdown cells. **e** Colony formation assay in DKC1-knockdown HepG2 cells. **f** CCK-8 assays showing the effect of DINAP on restoring cell proliferation in KO-DINAP cells. **g** Colony formation assay in KO-DINAP HepG2 cells with restoration of DINAP expression. **h** The protein level of DKC1 was examined in DKC1-

overexpressing wild-type and DINAP-KO HepG2 cells. The pcDNA-3.1 plasmid was used as the vector. **i** A working model of DINAP. NapRNA DINAP interact with DKC1 to promote cell proliferation by maintaining DKC1 protein stability in HepG2 cells. The values in **a-g** are mean ± SEM of 3 independent experiments. Two-sided t-test.Representative images of crystal violet staining of cells in **b**, **e** and **g** are shown. Colonies were counted in three independent experiments by ImageJ. Source data are provided as a Source Data file.
