# Supplementary material for: NAP-seq reveals multiple classes of structured noncoding RNAs with regulatory functions
Source: Nat Commun. 2024 Mar 18;15:2425. doi: 10.1038/s41467-024-46596-y (PMC10948791; doi:10.1038/s41467-024-46596-y)
Supplement: Supplementary file 3 — Description of Additional Supplementary Files [file 41467_2024_46596_MOESM3_ESM.pdf]

Description of Additional Supplementary Files

Supplementary Data Legends

Supplementary Data 1. Library list.

Supplementary Data 2. NapRNAs identified by NAP-seq.

Supplementary Data 3. NapRNAs which were differentially expressed.

Supplementary Data 4. Histone modification patterns of napRNAs

Supplementary Data 5. RepRNAs identified by NAP-seq.

Supplementary Data 6. SliRNAs identified by NAP-seq.

Supplementary Data 7. Snotrons identified by NAP-seq.

Supplementary Data 8. MisRNAs identified by NAP-seq.

Supplementary Data 9. List of primer and probe sequences
