# Supplementary material for: NAP-seq reveals multiple classes of structured noncoding RNAs with regulatory functions
Source: Nat Commun. 2024 Mar 18;15:2425. doi: 10.1038/s41467-024-46596-y (PMC10948791; doi:10.1038/s41467-024-46596-y)
Supplement: Supplementary file 13 — Reporting Summary [file 41467_2024_46596_MOESM13_ESM.pdf]

nature portfolio

Corresponding author(s): Jianhua Yang
Last updated by author(s): Feb 18, 2024

Reporting Summary

Nature Portfolio wishes to improve the reproducibility of the work that we publish. This form provides structure for consistency and transparency in reporting. For further information on Nature Portfolio policies, see our Editorial Policies and the Editorial Policy Checklist.

Statistics

For all statistical analyses, confirm that the following items are present in the figure legend, table legend, main text, or Methods section.

n/a	Confirmed	
☐	☒	The exact sample size (*n*) for each experimental group/condition, given as a discrete number and unit of measurement
☐	☒	A statement on whether measurements were taken from distinct samples or whether the same sample was measured repeatedly
☐	☒	The statistical test(s) used AND whether they are one- or two-sided<br>*Only common tests should be described solely by name; describe more complex techniques in the Methods section.*
☒	☐	A description of all covariates tested
☒	☐	A description of any assumptions or corrections, such as tests of normality and adjustment for multiple comparisons
☐	☒	A full description of the statistical parameters including central tendency (e.g. means) or other basic estimates (e.g. regression coefficient) AND variation (e.g. standard deviation) or associated estimates of uncertainty (e.g. confidence intervals)
☐	☒	For null hypothesis testing, the test statistic (e.g. *F*, *t*, *r*) with confidence intervals, effect sizes, degrees of freedom and *P* value noted<br>*Give P values as exact values whenever suitable.*
☒	☐	For Bayesian analysis, information on the choice of priors and Markov chain Monte Carlo settings
☒	☐	For hierarchical and complex designs, identification of the appropriate level for tests and full reporting of outcomes
☐	☒	Estimates of effect sizes (e.g. Cohen's *d*, Pearson's *r*), indicating how they were calculated

Our web collection on statistics for biologists contains articles on many of the points above.

Software and code

Policy information about availability of computer code

Data collection	High-throughput sequencing data was collected by Illumina X-ten sequencer. IrNorthern blotting images were collected by Odyssey software v3.0. ZEN lite 3.1 from ZEISS microscopy was used to acquire cell images. The amplification signal of qPCR data was acquired by QuantStudio Real-time PCR software v1.7.2. Images of agarose gel were collected by Uvtech1D.
Data analysis	Cutadapt v2.8; STAR 2.7.0; minimap2 v2.23; bedtools v2.17.0; limma v3.5.0; pheatmap v1.0.10; deepTools v3.5.1; R-scape v1.5.16; R2R v1.0.6; RNAz v2.1; Clustal W v2.1; Mfuzz v2.54.0; ggseqlogo; clusterProfiler; IGV v2.8.2;cutNapAdapter(https://github.com/junhong-huang/cutNapAdapter); napSeeker(https://github.com/junhong-huang/napSeeker);Perl scripts(https://github.com/junhong-huang/NAP-seq-Perl-scripts)

For manuscripts utilizing custom algorithms or software that are central to the research but not yet described in published literature, software must be made available to editors and reviewers. We strongly encourage code deposition in a community repository (e.g. GitHub). See the Nature Portfolio guidelines for submitting code & software for further information.

Data

Policy information about availability of data

All manuscripts must include a data availability statement. This statement should provide the following information, where applicable:

- Accession codes, unique identifiers, or web links for publicly available datasets
- A description of any restrictions on data availability
- For clinical datasets or third party data, please ensure that the statement adheres to our policy

Datasets that support the findings of this study are available in Source Data/Supplementary Data.

Software are available on the Github: napSeeker (https://github.com/junhong-huang/napSeeker), DOI: 10.5281/zenodo.10657490 and cutNapAdapter (github, https://github.com/junhong-huang/cutNapAdapter), DOI: 10.5281/zenodo.10670898. The Perl scripts are available in our Github: https://github.com/junhong-huang/NAP-seq-Perl-scripts, DOI: 10.5281/zenodo.10670900. All sequencing data that support the findings of this study have been deposited in NCBI's Gene Expression Omnibus (GEO). The NAP-seq data generated in this study have been deposited in the NCBI Gene Expression Omnibus (GEO) under accession code GSE192632 (https://www.ncbi.nlm.nih.gov/geo/query/acc.cgi?acc=GSE192632). The RNA-seq, CAP-seq, NAP-SHAPE-MaP, nanopore NAP-seq-TGS data generated in this study have been deposited in the NCBI GEO under accession code GSE228168 (https://www.ncbi.nlm.nih.gov/geo/query/acc.cgi?acc=GSE228168). GSE88089 for total RNA-seq, ENCSR000CRX for small RNA-seq and GSE160887 for PEN-seq in HepG2 cells, which are public data. There is no restriction on data availability.

Research involving human participants, their data, or biological material

Policy information about studies with human participants or human data. See also policy information about sex, gender (identity/presentation), and sexual orientation and race, ethnicity and racism.

Reporting on sex and gender	n/a
Reporting on race, ethnicity, or other socially relevant groupings	n/a
Population characteristics	n/a
Recruitment	n/a
Ethics oversight	n/a

Note that full information on the approval of the study protocol must also be provided in the manuscript.

Field-specific reporting

Please select the one below that is the best fit for your research. If you are not sure, read the appropriate sections before making your selection.

☒ Life sciences ☐ Behavioural & social sciences ☐ Ecological, evolutionary & environmental sciences

For a reference copy of the document with all sections, see nature.com/documents/nr-reporting-summary-flat.pdf

Life sciences study design

All studies must disclose on these points even when the disclosure is negative.

Sample size	The numbers of biological replicates for the experiment are indicated in the figure legends. For NAP-seq-NGS, three independent experiments were conducted for HepG2, HEK293T, U87, and four different stages of differentiation of C2C12 cells, respectively. Additionally, NAP-seq-TGS was independently performed three times for HepG2 cells and C2C12 myoblasts. Three independent experiments of NAP-SHAPE-Map were carried out for HepG2 cells. CAP-seq analysis and polyA-selected RNA-seq analysis in HepG2 cells were each performed at least two times. PolyA-selected RNA-seq analysis during four differentiated stages of C2C12 cells were conducted for three times. All images of polyacrylamide gels, agarose gels and northern blots are representative of at least two biological replicates. For all experiments, no sample size calculation was performed. Sample sizes were chosen empirically based on previously published work, knowledge of intra-group variation and expected effect sizes, or based on discussions with experts in their respective fields. These sample sizes were sufficient for statistical analysis.
Data exclusions	No data were excluded from analysis.
Replication	All experiments were performed in at least triplicate unless otherwise stated. All attempts at replication were successful.
Randomization	For all experiments, cells or samples were randomly assigned to control or treatment groups.
Blinding	For all experiments, blinding is not applicable as the quantitative analyses performed are not subject to human bias.

Reporting for specific materials, systems and methods

We require information from authors about some types of materials, experimental systems and methods used in many studies. Here, indicate whether each material, system or method listed is relevant to your study. If you are not sure if a list item applies to your research, read the appropriate section before selecting a response.

Materials & experimental systems

n/a	Involved in the study
☐	☒ Antibodies
☐	☒ Eukaryotic cell lines
☒	☐ Palaeontology and archaeology
☒	☐ Animals and other organisms
☒	☐ Clinical data
☒	☐ Dual use research of concern
☒	☐ Plants

Methods

n/a	Involved in the study
☒	☐ ChIP-seq
☒	☐ Flow cytometry
☒	☐ MRI-based neuroimaging

Antibodies

Antibodies used

1)anti-DKC1(for WB) rabbit, Abcam, Cat#ab156877, No clone#, Lot#GR117254-13, 1:2000
2)anti-DKC1 (for RIP and IF), mouse, Santa Cruz, Cat# sc-373956, No clone#, Lot#B2522
3)anti-MHC (for WB), mouse, R&D Systems, Cat#MAB4470, clone#MF20, Lot#CAE10416071, 1:4000
4)anti-Mef2c (for WB), rabbit, Proteintech, Cat#10056-1-AP, No clone#, Lot#00071848, 1:1000
5)anti-GAPDH (for WB), mouse, Proteintech, Cat#10494-1-AP, clone#1E6D9, lot#10013030, 1:5000
6)anti-FBL (for WB), mouse, Proteintech, Cat#66985-1-Ig, clone# 3A9E8, Lot#10008669, 1:2000
7)anti-β-Tubulin (for WB), rabbit, Cell Signaling Technology, Cat#2146, No clone#, Lot#4, 1?5000
8)anti-FLAG, mouse (for RIP), Proteintech, Cat#66008-2-Ig, clone#1E7B4, lot#10019027
9)anti-FBL (for IF), rabbit, Bethyl, Cat#A303-891A, No clone#, lot#1
10)anti-SC35 (for IF), mouse, Abcam, Cat#ab11826, No clone#, lot#GR3410532-1, 1:250
11)Alexa-Fluor 488 donkey, rabbit (for IF), ThermoFisher, Cat#A21206, No clone#, lot#2156521, 1:500
12)Alexa-Fluor 594 donkey, mouse (for IF), ThermoFisher, Cat#A21203, No clone#, lot#1820027, 1:500
13)IgG (for RIP), mouse, Proteintech, Cat# B900620, No clone#, Lot#20030599.

Validation

All vendors showed validation data for antibodies on their websites (including Western blot, IF, IP etc.).Manufacturer's Website: 1)anti-DKC1(cited in 2 publications: https://www.abcam.com/en-hk/products/primary-antibodies/dkc1-dyskerin-antibody-epr10399-ab156877#). In addition, we had validated this antibody in this study by DKC1-specific shRNAs. 2)anti-DKC1 (cited in 23 publications: https://www.scbt.com/p/dyskerin-antibody-h-3). In addition, we had validated this antibody in this study by DKC1-specific shRNAs. 3)anti-MHC (cited in 95 publications: https://www.rndsystems.com/cn/products/myosin-heavy-chain-antibody-mf20_mab4470#product-citations). 4)anti-Mef2c (cited in 39 publications: https://www.ptglab.com/products/MEF2C-Antibody-10056-1-AP.htm). 5)anti-GAPDH (cited in 6314 publications: https://www.ptglab.com/products/GAPDH-Antibody-60004-1-Ig.htm). 6)anti-FBL (cited in 3 publications: https://www.ptgcn.com/products/FBL-Antibody-66985-1-Ig.htm). 7)anti-β-Tubulin (cited in 963 publications: https://www.cellsignal.com/products/primary-antibodies/b-tubulin-antibody/2146). 8)anti-FLAG (cited in 73 publications: https://www.ptglab.com/products/Flag-tag-Antibody-66008-2-Ig.htm). In addition, we had performed RIP-WB to validate the performance of this antibody in this study. 9)anti-FBL (cited in 5 publications: https://www.fortislife.com/products/primary-antibodies/rabbit-anti-fibrillarin-antibody/BETHYL-A303-891#Citations). 10)anti-SC35 (cited in 107 publications: https://www.abcam.com/en-bn/products/primary-antibodies/sc35-antibody-sc-35-nuclear-speckle-marker-ab11826). 11)Alexa-Fluor 488 donkey, rabbit (cited in 4482 publications: https://www.thermofisher.cn/cn/zh/antibody/product/Donkey-anti-Rabbit-IgG-H-L-Highly-Cross-Adsorbed-Secondary-Antibody-Polyclonal/A-21206). 12)Alexa-Fluor 594 donkey, mouse (cited in 1522 publications: https://www.thermofisher.cn/cn/zh/antibody/product/Donkey-anti-Mouse-IgG-H-L-Highly-Cross-Adsorbed-Secondary-Antibody-Polyclonal/A-21203). 13)IgG (cited in 96 publications: https://www.ptglab.com/products/Mouse-IgG-B900620.htm).

Eukaryotic cell lines

Policy information about cell lines and Sex and Gender in Research

Cell line source(s)	HepG2 (SCSP-510), HEK293T (SCSP-502), U87(TCHu138) and C2C12 (SCSP-505) cells were purchased from the Cell Bank of the Chinese Academy of Sciences.
Authentication	Cells obtained from the Cell Bank of the Chinese Academy of Sciences relied on the company's certificates of analysis. No other independent authentication was performed.
Mycoplasma contamination	The cells were free of mycoplasma contamination based on the MycoBlue Mycoplasma Detector (Vazyme).
Commonly misidentified lines (See ICLAC register)	No misidentified cell lines that we know were used for this study.

Plants

Seed stocks	n/a
Novel plant genotypes	n/a
Authentication	n/a
